# Anomalous Glassy Thermal Conductivity in a Perovskite Bismuthate Induced by Structural Dynamic Instability

**DOI:** 10.1002/advs.202502379

**Published:** 2025-06-04

**Authors:** Alexandre Henriques, Mariana S. L. Lima, Gøran J. Nilsen, Matthias J. Gutmann, Steffen Wirth, Walber H. Brito, Valentina Martelli

**Affiliations:** ^1^ Institute of Physics University of São Paulo São Paulo 05508‐090 Brazil; ^2^ ISIS Neutron and Muon Source Didcot Oxfordshire OX11 0QX UK; ^3^ Max Planck Institute for Chemical Physics of Solids D‐01187 Dresden Germany; ^4^ Department of Physics Federal University of Minas Gerais Belo Horizonte 31270‐901 Brazil; ^5^ Department of Physics and Astronomy Rutgers University Piscataway New Jersey 08854 USA

**Keywords:** BaBiO_3_, glass‐like thermal conductivity, perovskites, unconventional superconductivity

## Abstract

Unraveling the underlying mechanisms at the origin of ultra‐low thermal conductivity in pristine crystals is of central relevance to designing new functional materials. Here, a study of thermal conductivity κ(*T*) in an extended temperature range (1.5–400 K) is reported in the single‐crystalline bismuthate perovskite BaBiO_3_. The measured κ(*T*) shows an anomalous glass‐like behavior with a ≈ *T* ^2^ dependence at low temperatures and a plateau in a wide temperature range from about 20 to 260 K, surprisingly recovering the expected downturn for a crystal only for *T* > 300 K. The measured room temperature κ ≈ 1.6 W m^−1^ K^−1^ agrees with the calculated value, including three‐phonon and isotope disorder scattering. However, the departure of the experimental from the calculated κ(*T*) for *T* < 300 K, at the onset of the plateau, indicates that an additional scattering mechanism comes into play at lower temperatures. It is proposed that a tunneling two‐level system associated with the BiO_6_ octahedra rotation offers an additional phonon scattering mechanism and may explain the observed κ(*T*) suppression. The findings may have significant implications for the pairing mechanism and unconventional superconductivity of doped‐BaBiO_3_ aside from suggesting it as a candidate building block for functional heterostructures.

## Graphical abstract

Unraveling the underlying mechanisms at the origin of ultra‐low thermal conductivity in pristine crystals is of central relevance to designing new functional materials. Single crystals of the candidate functional material BaBiO_3_ show an anomalous glassy thermal conductivity, which reveals the role of strong anharmonicity and additional scattering mechanism associated with a dynamic structural instability and a putative tunneling two‐level system. [Refs in Figure: SrTiO_3_ (Martelli et al. PRL 2018), SiO_2_ (Cahill et al. PRB 1992)]

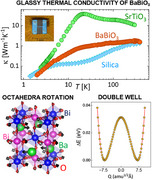

## 1 Introduction

The search for materials with reduced thermal conductivity is an active frontier of investigation. The engineering of interfaces, superlattices, and the synthesis of solids with weak chemical bonds^[^ ^1^, ^2^, ^3^, ^4^, ^5^ ^]^ have produced low‐thermal conductivity materials with great potential for thermoelectric and barrier coating applications.^[^ ^6^ ^]^ Materials that display poor heat conduction while simultaneously presenting a pristine crystalline structure have recently attracted attention as potential platforms for controlled fine‐tuning of the electronic properties.^[^ ^7^ ^]^ Concomitantly, the observation of highly reduced heat conduction in perfect crystals calls for further theoretical efforts to reveal and manipulate the underlying physical mechanisms. These features have been observed recently in a handful of compounds, and the heat conduction behavior of such materials is generally labeled as glass‐like, referring to the reduced thermal conductivity and their temperature dependence being similar to those typically observed in glasses.^[^ ^3^, ^8^ ^]^

The main heat carriers in perfectly insulating crystals are phonons, bosonic quasiparticles describing quantized lattice vibrations. Upon cooling, the thermal conductivity of a crystal is expected to increase from its room temperature value, attaining a maximum (peak thermal conductivity) at intermediate temperatures (≈ 0.1Θ_D_,^[^ ^9^ ^]^ where Θ_D_ is the material's Debye temperature). This is because the momentum loss due to phonon–phonon scattering (Umklapp events) is reduced due to the decrease of the phonons' energy. When the temperature is further reduced, the phonon population becomes scarce, and κ(*T*) starts to decrease, displaying a *T* ^3^ power law at very low temperatures in agreement with the Debye model.^[^ ^10^ ^]^

In glasses and amorphous compounds, the arrangement of atoms lacks spatial long‐range order, and several typical signatures are universally observed in the temperature evolution of thermal conductivity. In particular, the thermal conductivity shows a monotonic increase at high‐*T*, typically reaching a value of the order of 1 W m^−1^ K^−1^ around room temperature, a plateau at intermediate temperature (*T* ≈ 10 K), and a *T* ^2^ power law at cryogenic temperatures.^[^ ^11^ ^]^ The low‐temperature features were early interpreted within phenomenological models, such as the tunneling two‐level system (TTLS) model.^[^ ^12^, ^13^, ^14^ ^]^ A TLLS is ascribed to the presence of atoms or groups of atoms in metastable states, described by a double‐well potential, and acts as a scattering centers for the traveling heat. The high‐*T* steady increase can be understood in terms of the energy of the localized vibrational modes, which are enhanced by raising the temperature and responsible for the heat transfer.^[^ ^15^ ^]^

If a pristine insulating single crystal with a regular structure possesses such a glass‐like behavior, a question arises about what mechanisms can cause this to happen. Single‐crystalline materials showing features of a glassy thermal conductivity are rare. Still, for those found so far, a magnetic instability, magnetic frustration, or phase instability (ferro‐ or antiferro‐electric transition) were proposed to be at the base of the possible scattering mechanisms that would generate a TTLS in analogy to glasses.^[^ ^3^, ^8^, ^16^, ^17^, ^18^, ^19^, ^20^ ^]^ Away from the very low‐temperature range, the interpretation becomes more complex. Some materials do not show a clear plateau followed by a steady increase (ex.: BaTiS_3_^[^ ^8^ ^]^ or Cs_3_Bi_2_I_6_Cl_3_^[^ ^3^ ^]^) as expected for a glass while some do (ex.: NaNbO_3_^[^ ^16^ ^]^ or EuTiO_3_^[^ ^20^ ^]^). In that temperature range, multiple effects of phonon scattering concomitantly contribute to the resulting thermal conductivity, making the identification of the dominant mechanism often an unsolvable puzzle. Furthermore, unavoidable structural disorder and stoichiometric deviation can play an important role, making a thorough inspection of the compound's quality indeed a paramount to discern when they display unusual glass‐like behavior due to inherent phase instability.

Many‐body theories combined with first‐principles and molecular dynamics (MD) methods have been used to advance the current understanding of the scattering mechanisms of heat in solids.^[^ ^21^, ^22^, ^23^ ^]^ However, they are very dependent on the determination of high‐order interatomic force constants and/or parametrization of force fields, which can be very computationally demanding for low‐symmetry crystal structures and/or with large unit cells.^[^ ^24^ ^]^ More recently, novel theoretical approximations were proposed for a quantitative description of the thermal conductivity of materials from first‐principles.^[^ ^25^, ^26^, ^27^, ^28^, ^29^ ^]^

In this work, we study the thermal properties of BaBiO_3_, which is a semiconducting, non‐magnetic perovskite oxide, and parent compound of high‐*T*_*c*_ bismuthate‐based cubic superconductors Ba_1 − *x*_K_*x*_BiO_3_.^[^ ^30^, ^31^ ^]^ Known since the 1970s, BaBiO_3_ has regained attention as a promising candidate for building new functional heterostructures motivated by the prediction that they may host a topological insulating state when electron‐doped.^[^ ^32^, ^33^, ^34^, ^35^, ^36^ ^]^ Only recently, the magnitude of the superconducting transition temperature was calculated within a picture of strong electron‐phonon coupling enhanced by electronic correlations.^[^ ^37^, ^38^ ^]^ The interplay between the electron–phonon coupling with charge order or bipolaronic superconductivity have been recently discussed in literature.^[^ ^39^, ^40^ ^]^ When not doped, BaBiO_3_ displays a monoclinic symmetry at room temperature (I2/m space group), with a combination of tilting and breathing distortions of the BiO_6_ octahedra. Lowering the temperature, it smoothly reaches a monoclinic phase with different symmetry (P2_1_/n), which also exhibits breathing and tilting distortions. At high temperatures, for *T* > 800 K, these distortions are suppressed and the system recovers a cubic phase ($\mathrm{Fm}\bar{3}m$).^[^ ^41^ ^]^ In this context, the unsolved question on the origin of the ground state of BaBiO_3_ reemerged with a refreshed relevance.^[^ ^42^, ^43^, ^44^, ^45^ ^]^ Although thermal transport can reveal important information about the insulating ground state and the physics of heat carriers, thermal conductivity in BaBiO_3_ remained unexplored except for a recent study on polycrystalline samples.^[^ ^46^ ^]^

Here, we report on our experimental investigation of thermal conductivity and specific heat of high‐quality BaBiO_3_ commercially available single crystals (SurfaceNet). The thermal conductivity of BaBiO_3_ reveals an anomalous combination of features reminiscent of both a crystal and a glass depending on the temperature range: *(i)* it presents a low value of κ_300K_ ≈ 1.6 W m^−1^ K^−1^ with an increasing behavior upon cooling typical of a crystalline solid at high temperatures (300 K <*T* < 400 K); *(ii)* it shows a downturn at *T* ≈ 260 K followed by an extended region of constant mild decrease, which we call the “plateau”, within the *T*‐range of approximately 20 – 260 K; finally, *(iii)* it displays *k*(*T*) ∼ *T* ^2^ at low temperatures (*T* < 5 K). Specific heat experiments at very low temperatures reveal the presence of a quasi‐linear contribution for *T* < 1 K. From the theoretical side, our calculated κ(*T*) agrees well with the experimental data for *T* > 300 K, in the region where a behavior typical of a crystal is observed. At the onset of the plateau, the theoretical κ(*T*) ∼ *T* ^−0.9^ shows a departure from the experiments. We discuss the possible origin of this set of observations in the light of our first‐principles calculations and a putative TTLS. We propose that an unusual structural dynamic instability associated with the monoclinic distortions may be the source of important scattering mechanisms to explain the glassy behavior and the lattice anharmonicity, in addition to typical mechanisms usually ascribed to reduced thermal transport in crystalline materials.

## 2 Anomalous Glassy Thermal Conductivity

**Figure 1:**
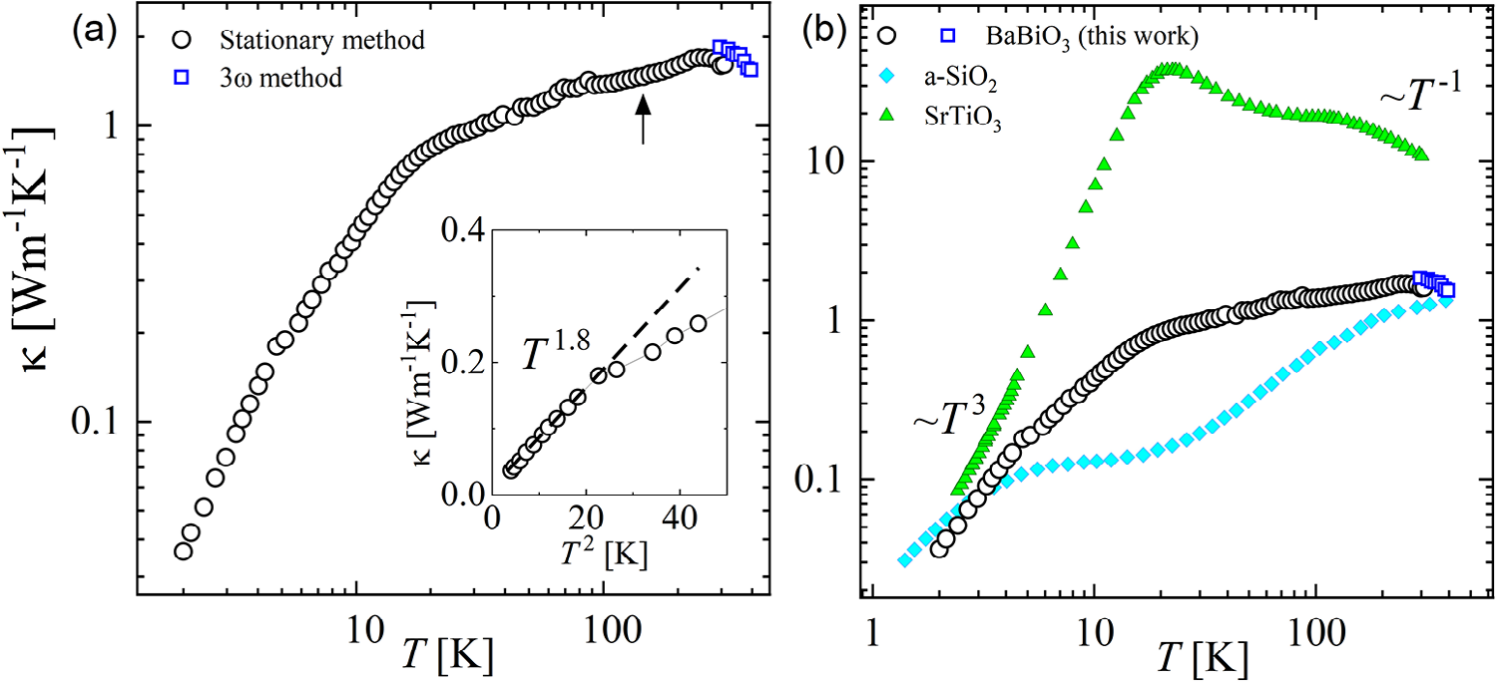
Thermal conductivity of BaBiO_3_. a) κ(*T*) measured by two experimental methods. It displays a plateau in an extended temperature region. The arrow indicates the position of the structural transition between the two monoclinic phases. κ(*T*) does not detect any anomaly at the transition. The inset shows a zoom of the κ(*T*) into the low‐temperature region displaying a *T* ^2^ dependence at *T* < 5 K. The dashed line is a guide for the eyes. b) Comparison of thermal conductivity of BaBiO_3_ (this work) with SrTiO_3_^[^ ^47^ ^]^ and a‐SiO_2_ (Silica).^[^ ^48^ ^]^

The thermal conductivity of BaBiO_3_ in the 2 ‐ 400 K temperature range is shown in **Figure** **1**a. Figure 1b displays the comparison with representative SrTiO_3_ (crystalline insulator) and a‐SiO_2_ (amorphous). The room temperature $\kappa_{\text{300K}}\approx \;1.6$ W m^−1^ K^−1^ is close to the thermal conductivity of a‐SiO_2_ and about one order of magnitude lower than in other insulating perovskite oxides, e.g., ≈10 W m^−1^ K^−1^ for SrTiO_3_^[^ ^47^ ^]^ and 11 W m^−1^ K^−1^ for KTaO_3_.^[^ ^16^ ^]^ The mismatch in the room‐*T* value for the measurements obtained with the two experimental techniques, κ ≈ 1.6 W m^−1^ K^−1^ (black circles in Figure 1a) and κ ≈ 1.8 W m^−1^ K^−1^ (blue squares in Figure1b) falls within the experimental resolution and errors (≈ 9–12%).

Upon cooling from 400 K, κ(*T*) increases, as expected for a crystal at high temperatures. The high‐*T* decrease in κ(*T*) is also shown in Figure 5. We emphasize that BaBiO_3_ undergoes a structural phase transition around *T* > 430 K,^[^ ^41^ ^]^ which is above the temperature range investigated in this work. However, that rise is soon aborted around *T* ≈ 260 K, when the curve begins an unexpected decrease. Further reducing the temperature, the value of thermal conductivity remains almost constant in a wide temperature range, displaying a mild reduction down to κ ≈ 1.0 W m^−1^ K^−1^ at around *T* ≈ 20 K, a region of weak temperature dependence, which we call “plateau”. Within the plateau, a smooth change of slope can be observed around *T* ≈ 70 K when plotted on a linear scale, as shown in Figure 5 (black arrow). This change of slope reveals that a very efficient scattering effect is activated, drastically suppressing the phonon‐phonon scattering that typically rules κ(*T*) ∼ *T* ^−1^ at this temperature range in a crystal and that should instead lead to a steady increase upon cooling up to a typical peak as observed in SrTiO_3_ (Figure 1b). The black arrow in Figure 1a indicates the approximate temperature where the structural phase transition between monoclinic‐I (P2_1_/n) and monoclinic‐II (I2/m) is expected. Unsurprisingly, κ(*T*) itself does not seem to be affected by that transition since that was shown to be a second‐order transition with a smooth change of the lattice parameters.^[^ ^41^, ^49^ ^]^

**Figure 2:**
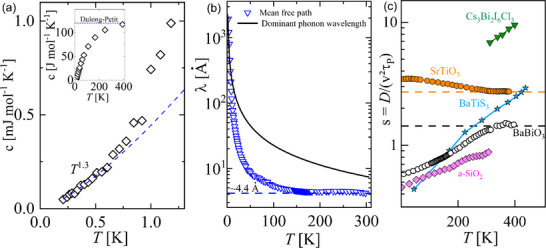
Specific heat, apparent mean free path, and thermal diffusivity of BaBiO_3_. a) Quasi‐linear‐in‐*T* behavior at very low temperatures. The inset shows the specific heat from 0.2 to 400 K. The blue line indicates the Dulong‐Petit value, *c* → 15*R*. b) The apparent mean free path computed from λ = 3κ/(*cv*). The black line represents the wavelength of the dominant phonon calculated by λ = *hv*/*k*_*B*_ *T*. The blue dotted line shows the value λ approaches at high‐*T* ≈ 4.4Å. c) Saturation of the thermal diffusivity *D* = κ/*C* toward the expected lower bound set by sound velocity (*v*) and the planckian time (τ_*P*_ = ℏ/*k*_*B*_ *T*). The horizontal dashed lines (orange and blue) represent the lower bound for SrTiO_3_ and BaBiO_3_, respectively. BaBiO_3_ approaches the limit close to room temperature. Θ_D_ ≈ 513 K for SrTiO_3_.^[^ ^52^ ^]^ Data of Cs_3_Bi_2_I_6_Cl_3_, BaTiS_3_, and a‐SiO_2_ (Silica) were extracted from^[^ ^3^, ^8^ ^]^ and,^[^ ^11^ ^]^ respectively.

**Figure 3:**
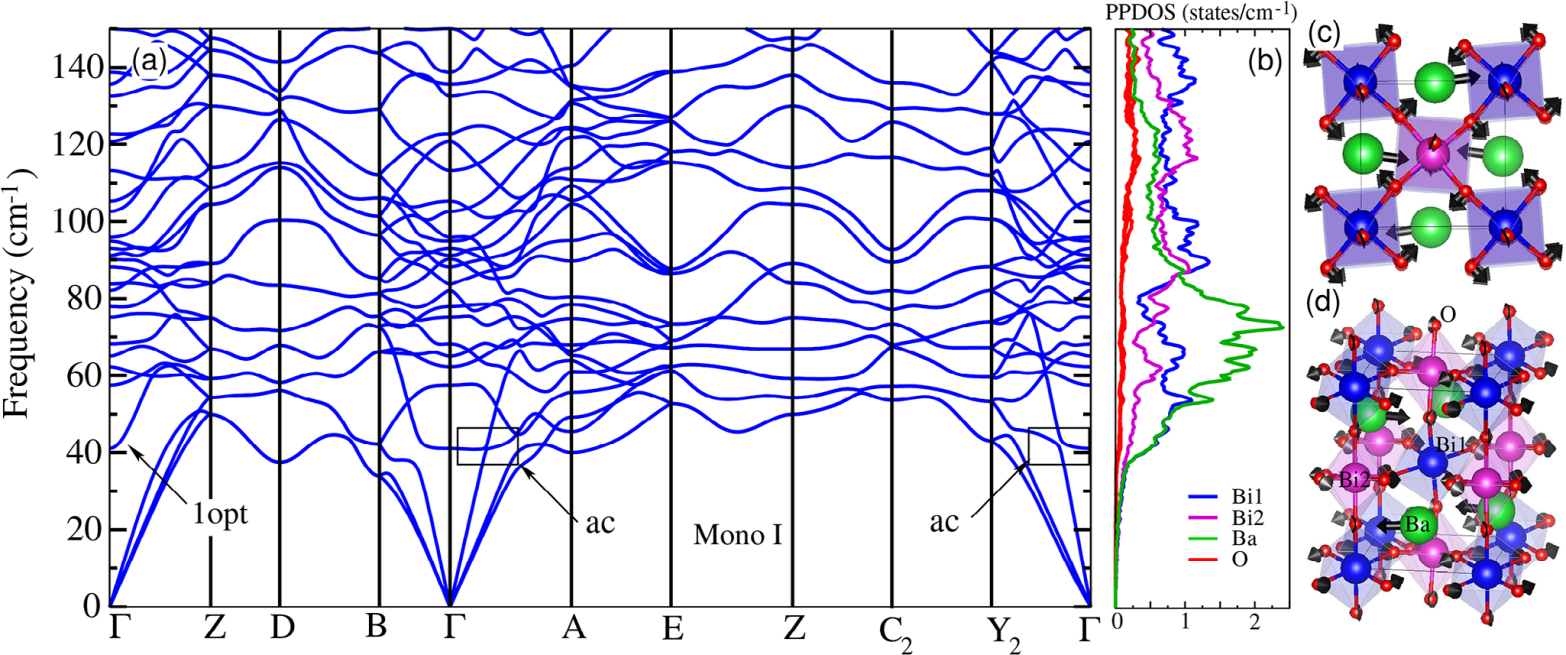
Phonon dispersion of BaBiO_3_ and the low‐energy optical modes: a) DFT obtained phonon dispersion of monoclinic I phase (P21/n). The black boxes and the low‐energy optical phonon mode indicate the avoided‐crossing (ac) regions in the phonon dispersion (1opt). In (b) we show the projected phonon density of states (PPDOS), for Bi1, Bi2, Ba, and O atoms shown in panel (d). In (c) and (d) we show the real space representation of the eigenvector of the first optical (1opt) phonon at the Γ point. Blue and violet spheres represent Bi1 and Bi2 atoms, respectively. Green and red spheres represent Ba and O atoms.

**Figure 4:**
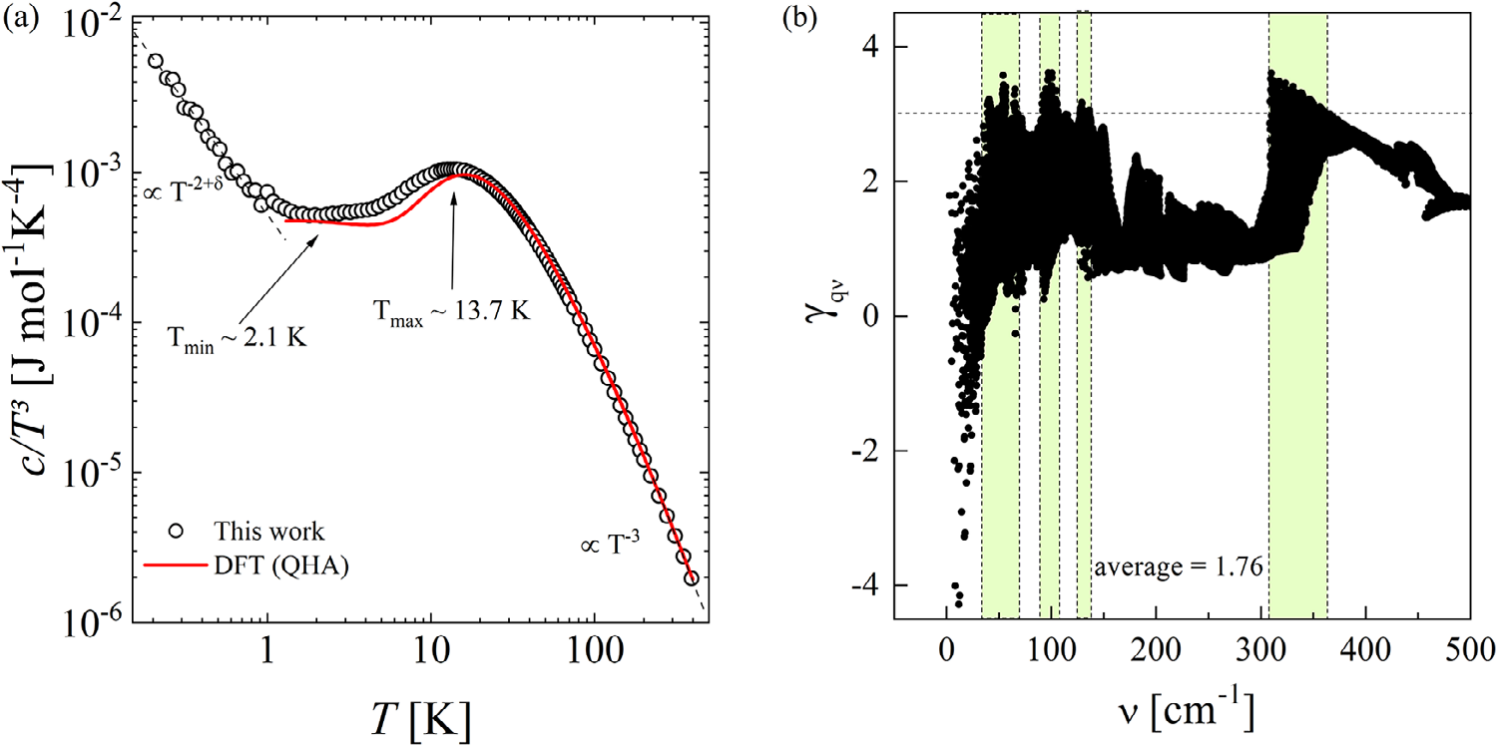
Anharmonicity in BaBiO_3_: a) *c*/*T* ^3^ plot of BaBiO_3_ specific heat data. A local minimum at *T*_min_ = 2.1 K is seen, while an excess over the Debye modes is attributed to the Boson‐like peak at *T*_max_ = 13.7 K. The red line represents the specific heat obtained within the quasi‐harmonic approximation ‐ DFT(QHA). b) Calculated mode Grüneisen parameters as a function of frequency. Green shaded areas indicate where γ_**q**ν_ > 3.

**Figure 5:**
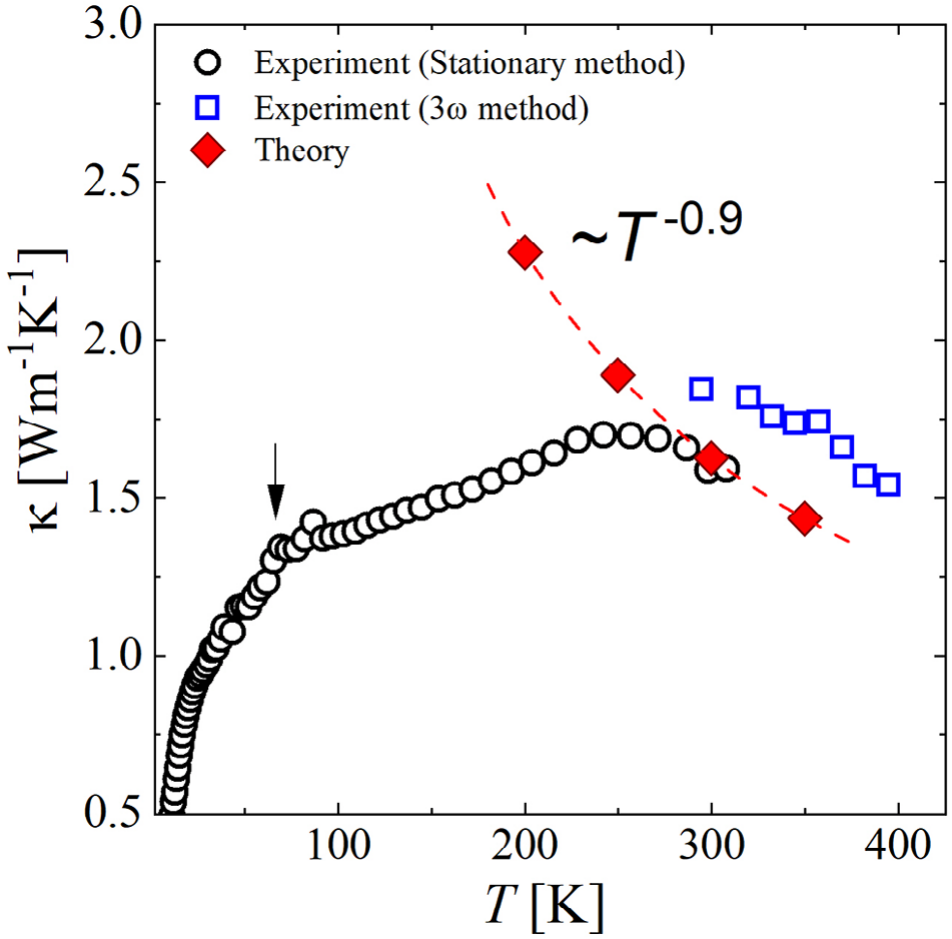
Measured and calculated κ(*T*): Comparison between the experimental values of κ(*T*) obtained by two different techniques and the calculated values obtained within the mode‐coupling approximation. A reasonable agreement is shown down to the onset of the plateau, where a departure from the data is observed. The arrow ( *T* ≈70 K) points to a change in the slope within the plateau.

In the lowest temperature range, *T* < 5 K, a thermal conductivity κ(*T*) ∼ *T* ^2 − δ^, with δ ≈ 0.2, is found. These findings are different from what is expected for a crystal according to a simplified kinetic approach to a phonon gas—see inset of Figure 1a. There, the thermal conductivity in an insulating crystal is accounted for by $\kappa =\frac{1}{3}v\lambda c$, where *c* is the specific heat, λ is the mean free path and *v* the sound velocity. According to this picture, a resulting *T* ^3^ power law is expected at low temperatures due to a weak variation of the elastic constants (and sound velocity), a *T* ^3^ dependence of Debye specific heat and a saturating mean free path due to the large phonon wavelength compared with the domains or sample size (Figure 1b). In several other complex oxides, the missing *T* ^3^ power law can be ascribed to extrinsic reasons (disorder) or intrinsic reasons (phase instability); however, in the case of BaBiO_3_, both the high‐quality of the samples, proven by SXD and the absence of phase instability of any kind suggest that a dynamic structural instability, in the analogy to glasses, could be the origin of an equivalent TTLS scattering centers for phonons.

Clearly, the temperature evolution of our thermal conductivity measurements points to the first experimental evidence of glass‐like behavior in single crystals of BaBiO_3_.

## 3 Specific Heat and Thermal Diffusivity

Our specific heat measurements *c*(*T*) are displayed in **Figure** **2**a. The curve approaches the Dulong‐Petit limit for *T* > 400 K (see inset). For the very low temperature range (*T* ⩽ 700 mK) of *c*(*T*), we can detect a super‐linear behavior of *c*∝*T* ^1 + δ^ with δ ≈ 0.3. As the electric resistivity of BaBiO_3_ is about 10^5^ Ω.cm,^[^ ^46^, ^50^, ^51^ ^]^ an electronic contribution can be safely neglected, and the linear term must be attributed to another contribution. A Schottky contribution could also lead to a linear‐in‐*T* specific heat at very low temperatures; however, to the best of our knowledge, BaBiO_3_ neither magnetically orders at low temperatures nor possesses magnetic impurities in the case of the crystals here investigated. Quasi‐linear specific heat *c* ≈ *T* ^1 + δ^ is also expected within the TTLS model proposed by Anderson and coworkers in the case of glasses.^[^ ^12^ ^]^ The latter could suggest that an analogous phonon scattering mechanism is present in BaBiO_3_ crystals, consistent with the observation in κ(*T*). It is worth mentioning that for any particle in a double‐well potential, the formalism leads to a linear‐in‐*T* specific heat, regardless of the potential's origin.

By computing the apparent mean free path λ = 3κ/(*cv*) (Figure 2b), we find that λ approaches a saturation value of ≈ 4.4 Å  (similar to the Bi‐Bi distance along the [001]‐crystallographic direction) already for *T* > 100 K up to room temperature (dashed blue line). The black solid line shown in Figure 2b represents the wavelength of the dominant phonon calculated by λ = *hv*/*k*_*B*_ *T*. The longitudinal sound velocity is computed based on our calculated elastic moduli (see Section S6, Supporting Information) obtained from our DFT calculations. Inspecting the thermal diffusivity *D* = *k*/*c*ρ, where ρ is the volumetric density, can provide further insights into this finding. Recently, the thermal diffusivity of insulating crystals at high temperatures was shown to never decrease below a lower boundary set by the Planckian time:^[^ ^47^, ^53^, ^54^, ^55^ ^]^ *D* = *sv* ^2^τ_*P*_ where τ_*P*_ = ℏ/*k*_*B*_ *T* and *s* is a dimensionless parameter that does not fall below 1. Thus, if one plots *s* = *D*/(*v* ^2^τ_*P*_) for a crystal, the curve will asymptotically reach a constant value *s* > 1 for temperatures close to the Debye temperature Θ_D_ due to the kinetic conductivity regime leading to *D*∝*T* ^−1^, as, e.g., in the case of SrTiO_3_ (Figure 2c). Behnia & Kapitulnik^[^ ^54^ ^]^ have phenomenologically observed that glasses also seem to respect the lower boundary of diffusivity, but only asymptotically (see a‐SiO_2_ in Figure 2c). If we compute *s* for Cs_3_Bi_2_I_6_Cl_3_ (Θ_D_ ≈ 80 K^[^ ^3^ ^]^) and BaTiS_3_ (Θ_D_ ≈ 600 K^[^ ^8^ ^]^), two recently reported pristine crystals showing glassy behavior,^[^ ^3^, ^8^ ^]^ we can see that *s* is far from saturation. A similar argument holds for Silica. However, with Θ_D_ ≈ 410 K, BaBiO_3_ reaches *s* → 1.5 as *T* approaches room temperature, displaying a consistent saturation up to 400 K. Thus, BaBiO_3_ recovers the expected behavior of a crystal, suggesting that the scattering mechanism determining the glass‐like conductivity loses strength for *T* > 260 K.

## 4 Phonon Dispersion, Low‐Frequency Optical modes and Grüneisen Parameters

To deepen our understanding of the phononic properties of BaBiO_3_, we carried out first principles calculations (details in Sections S5–S10, Supporting Information) to obtain the phonon dispersion of the monoclinic I (P21/n) structure. The monoclinic I phase is the stable structure at low temperatures followed by the monoclinic II phase, above 140 K. Both structures exhibit breathing and tilting distortions, with a minor difference regarding the rotation of BiO_6_ octahedra and the emergence of an extra inequivalent position of an oxygen atom.^[^ ^41^ ^]^ In **Figure** **3**a, we show the calculated phonon dispersion of monoclinic I phase. We observe *(i)* the existence of a low‐frequency optical mode, with ω ≈ 41 cm^−1^ at the Γ point, and a reasonable number of *(ii)* weakly dispersive modes centered between 40 and 80 cm^−1^. These modes are mostly due to the displacement of Ba atoms, as emphasized by the projected phonon density of states (PPDOS) (Figure 3b). Finally, we point out the *(iii)* avoided crossing points along *Y*_2_ – Γ and Γ − *A* directions (indicated by the arrows), which are fingerprints of significant acoustic‐optical (a‐o) phonon hybridization, as similarly observed in the cases of PbTe,^[^ ^56^ ^]^ and YbFe_4_Sb_12_.^[^ ^57^ ^]^ In the latter, it was found that these features are of central importance to the increase of three‐phonon scattering processes.

The corresponding real space representation of the eigenvector of the first optical mode at the Γ point is shown in Figure 3c,d, where one can notice the more significant contribution of the Ba atoms and the BiO_6_ octahedra rotation. These modes with Ba displacements are reminiscent of localized rattling‐like centers induced by loose Ba‐O bonds (see Section S6, Supporting Information). We can, therefore, attribute the presence of low‐lying optical phonon modes to the weakly bound Ba, which in turn are likely to couple with acoustic modes. Similar features were observed in the case of the Cs_3_Bi_2_I_6_Cl_3_ compound.^[^ ^3^ ^]^ These findings indicate that these low‐frequency optical modes can lead to additional scattering of acoustic phonons and, as a result, to the suppression of the thermal conductivity of BaBiO_3_.

Plotting the measured *c*/*T* ^3^ as a function of *T* reveals the presence of a peak at *T*_*peak*_ ≈ 13.7 K (**Figure** **4**a). We also show in the same figure the calculated lattice specific heat (in red) obtained within the quasi‐harmonic approximation (QHA) (see Section S8, Supporting Information). The emergence of a peak at 13.7 K is a clear mark of an excess of vibrational modes over the Debye density of states, indicating a deviation from a simple Debye model. In fact, that peak has generally been considered as indirect evidence of relevant localized vibrational modes (Einstein‐like modes,^[^ ^58^, ^59^, ^60^ ^]^ in agreement with the four Θ_*E*_ found in the specific heat analysis ‐ see Section S4, Supporting Information), in addition to the Debye density of states. Being ascribed to anything but acoustic modes, the peak can be interpreted in multiple ways depending on the material.^[^ ^61^ ^]^ For instance, it can indicate the existence of acoustic‐optical phonon coupling,^[^ ^62^, ^63^ ^]^ rattling modes,^[^ ^64^ ^]^ or can be even an indirect evidence of soft modes.^[^ ^65^ ^]^ In our case, the a‐o coupling is revealed by the excess of modes over the Debye density of states in the proximity of the peak in *c*/*T* ^3^, found in both calculations and experiment (see Section S4, Supporting Information) and the avoided crossings in the phonon dispersion. Moreover, the good agreement between the experimental and theoretical *c*(*T*)/*T* ^3^ curves for *T* > 1 K, highlights that the specific heat is well described in this limit by the lattice vibrations and its implicit anharmonicity. On the other hand, we stress that a quasi‐harmonic picture of BaBiO_3_ cannot capture the linear behavior seen in measured *c*/*T* ^3^ at low temperatures.

Microscopically, the strong reduction in thermal conductivity because of enhanced a‐o coupling was proposed elsewhere^[^ ^57^, ^66^ ^]^ as an elevation of Umklapp events via “acoustic” + “optical” → “ optical” at certain frequencies. It is worth noticing that the avoided crossings persist in the cubic phase *T* > 800 K, as shown by the calculated phonon spectral function (see Section S8, Supporting Information). This suggests they are intrinsic and not a reminiscent feature of the structural transitions. At this point, it is instructive to establish an analogy between the role of Ba‐O bonds in BaBiO_3_ and the typical behavior of guest atoms in filled skutterudites and clathrates.^[^ ^67^, ^68^, ^69^ ^]^ Recently,^[^ ^70^, ^71^, ^72^ ^]^ perovskites were demonstrated to present rattling as well, which all together leads to the same pieces of evidence (avoided‐crossing, Boson peak, high atomic displacement parameters of the guest atom, etc.) in multiple experiments. Often, there is an association of rattling with the concept of sufficient interstitial (guest‐cage) distance.^[^ ^64^, ^70^, ^71^ ^]^ Nonetheless, one cannot assume that rattling‐like mechanisms are a sufficient condition for glassy thermal conductivity, in particular, to explain the *T* ^2^ dependence at very low temperatures, since localized vibrations are expected to “freeze‐out” far below their characteristic temperatures.^[^ ^73^, ^74^ ^]^

Another important aspect associated with glass‐like features in the thermal transport is the lattice anharmonicity of a given material. To quantify the lattice anharmonicity of BaBiO_3_, we calculated the mode‐Grüneisen parameters. We mention that $\gamma_{q\nu }=-\frac{V}{\omega_{q\nu }}\frac{\partial \omega_{q\nu }}{\partial V}$, where **q** and ν are the wavevectors and the mode index, respectively. γ_**q**ν_ are important parameters to describe how the phonon frequencies shift under volume expansions/contractions (implicit anharmonicity), and were calculated using the derivative of the dynamical matrix performed within the finite difference method for three distinct volumes.^[^ ^75^ ^]^ In Figure 4b we show the calculated mode‐Grüneisen parameters as a function of frequency. Overall, we found positive Grüneisen parameters between 1 and 3.5 for the low‐energy optical modes, with an average of γ_*avg*_ = 1.76. As can be noticed in Figure 4b, modes with frequencies around 40 cm^−1^ present γ_**q**ν_ > 3, indicating a sizable *softness* of these optical modes with volume expansion, which can be induced upon warming. Similar large Grüneisen parameters are observed for modes centered around 52, 67, 97, and 311 cm^−1^ (green frequency intervals in Figure 4b). These findings indicate a reasonable anharmonicity of phonon modes in BaBiO_3_. We mention that the predicted mode shifts based on our calculated Grüneisen parameters can be underestimated (such as in silicon^[^ ^76^ ^]^), since we do not include high‐order force constants in our theoretical approximation. As a result, the softness of the modes in BaBiO_3_ can be even larger.

## 5 Theoretical Lattice Thermal Conductivity and Comparison with Experiments

Our previous theoretical analysis indicates that important acoustic‐optical coupling and anharmonicity are intrinsic to the lattice dynamics of BaBiO_3_, and that this results in a low thermal conductivity. To estimate how these features impact the transport of heat in pristine BaBiO_3_ single crystals, we calculate its lattice thermal conductivity at 200, 250, 300, and 350 K. These calculations were performed for the monoclinic II phase, which is the stable structure above 140 K. The lattice thermal conductivity was calculated within the mode‐coupling theory,^[^ ^28^, ^29^ ^]^ including third‐order anharmonicity (three‐phonon scattering) and the scattering from isotope disorder.^[^ ^77^ ^]^ Therefore, in our theoretical framework the scattering matrix is determined considering a phonon self‐energy written as Σ_*s*_(**q**, ω) = Δ_*s*_(**q**, ω) + *i*Γ_*s*_(**q**, ω), where the imaginary part of the self‐energy is given by $\Gamma_{s}\left(q,\omega \right)=\Gamma_{s}^{\left(3\right)}\left(q,\omega \right)+\Gamma_{s}^{iso}\left(q,\omega \right)$. More details can be found in Section S10 (Supporting Information). Within this formalism, we also include the off‐diagonal (coherence) contributions of the scattering matrix to the thermal conductivity tensor $\kappa_{\alpha \beta }=\kappa_{\alpha \beta }^{d}+\kappa_{\alpha \beta }^{od}$. For the diagonal contribution, the components of the scattering matrix correspond to the scattering rates. The off‐diagonal component takes into account the collective phonon contributions, which can be important for materials with complex structures.^[^ ^78^ ^]^ We also mention that when the off‐diagonal part is neglected, the employed approximation becomes equivalent to the phonon Boltzmann transport equation (PBTE).^[^ ^79^ ^]^

Our results for the directional average $\kappa_{\text{avg}}=\frac{1}{3}\left(\kappa_{xx}+\kappa_{yy}+\kappa_{zz}\right)$ are shown in red in **Figure** **5**, where one can notice a good agreement between the calculated and measured lattice thermal conductivity at high temperatures. At each temperature, we find a small difference between κ_*xx*_ and κ_*yy*_, while κ_*zz*_ is predicted to be slightly higher. At 300 K, for instance, we obtain κ_*xx*_ = 1.53 W m^−1^ K^−1^, κ_*yy*_ = 1.59 W m^−1^ K^−1^, and κ_*zz*_ = 1.76 W m^−1^ K^−1^. The obtained off‐diagonal contribution (coherence part) at 300 K is around 0.16 W m^−1^ K^−1^. The calculated κ_avg_(300K) = 1.63 W m^−1^ K^−1^, whereas the experimental values are 1.6 – 1.8 W m^−1^ K^−1^ (stationary/3ω method). Overall, we find that by including three‐phonon scattering and isotope scattering due to the natural isotopic distribution, our theoretical model yields low values for the lattice thermal conductivity of BaBiO_3_. According to our findings, the three‐phonon scattering is dominant and indicates that events related to acoustic‐optical phonon scattering are effective in reducing the thermal conductivity of BaBiO_3_ at higher temperatures. In addition, we find that κ(*T*) ∼ *T* ^−0.9^, similar to *T* ^−1^ dependence of systems with temperature independent interatomic force constants (IFCs). In such systems, as in our theoretical treatment, the temperature dependence of κ arises primarily from the phonon occupation. In the work of Klarbring and coworkers,^[^ ^80^ ^]^ a proper temperature dependence of the IFCs predicted a κ(*T*) ∼ *T* ^−0.5^ behavior for a lead‐free halide double perovskite. More importantly, one can notice that our theoretical approximation cannot reproduce the glass‐like behavior of the measured thermal conductivity. In particular, we observe a strong deviation between the theoretical and experimental values for *T* < 260 K, indicating that within this temperature range there are important scattering mechanisms which are neglected within our theoretical framework.

## 6 Putative TTLS and Its Effect at High Temperatures

Glass‐like thermal properties have been observed in a few single‐crystalline materials of proven quality, such as Cs_3_Bi_2_I_6_Cl_3_^[^ ^3^ ^]^ and BaTiS_3_.^[^ ^8^ ^]^ In the former, the glass‐like features were explained based on intrinsic anharmonicity and weakly dispersive optical phonon modes. In the latter, bimodal distribution, and the associated tunneling of Ti atoms in a shallow double‐well potential, were invoked to explain the unusual behavior. The existence of weakly dispersive optical modes and bimodal distributions of atoms in those structures calls for additional models beyond the simple TTLS, which, in particular, cannot explain the high‐temperature part of the thermal conductivity. The soft potential model (SPM)^[^ ^81^ ^]^ can be considered one example of extending the TTLS model. Nevertheless, in the case of BaTiS_3_, it is a remarkable finding that a dynamic structural instability has sizable effect on the thermal transport well beyond cryogenic temperatures,^[^ ^8^ ^]^ whereas TTLS have been typically identified as relevant scattering centers only at low temperatures.

In BaBiO_3_, both *k*(*T*) and *c*(*T*) show indications of glass‐like behavior, but two main questions arise. The first is on the origin of the putative TTLS in BaBiO_3_. The second question is whether those putative TTLS still play a role at higher temperatures, as in the case of BaTiS_3_, in addition to the other identified scattering sources. We recall that the plateau, where the thermal conductivity shows a very mild increase, extends from 20 up to 260 K, suggesting that the mechanisms at the base of this efficient phonon scattering become inefficient above that temperature threshold. We may attempt to interpret the findings by the transition energies of a ‘particle’ within the double‐potential well.

**Figure 6:**
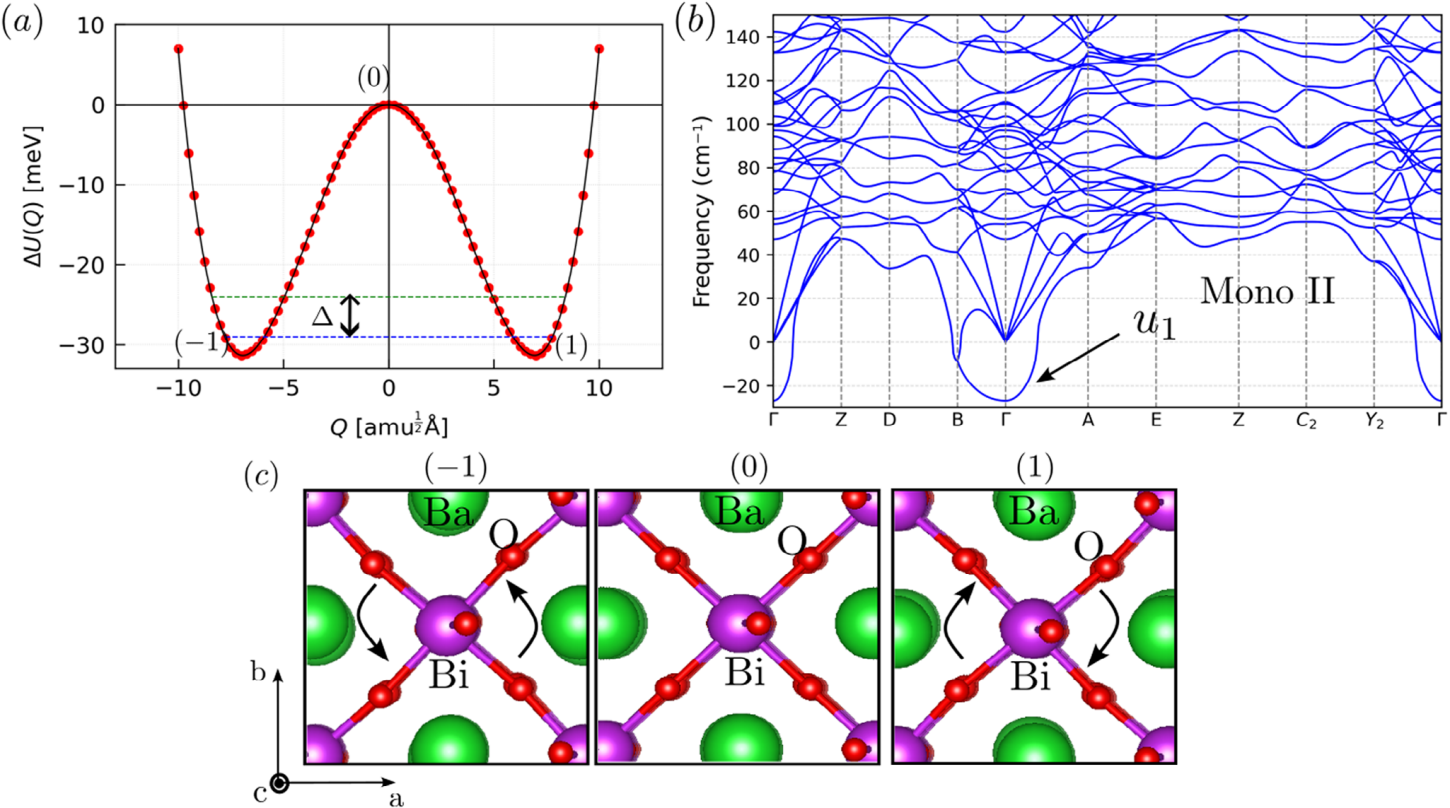
Putative TTLS in BaBiO_3_: a) Double‐well potential energy surface along the unstable acoustic phonon mode (*u*_1_) at Γ point. Δ indicates the energy splitting of a particle within this double‐well potential. (0), (− 1), and (1) correspond to the different configurations shown in (c). b) DFT obtained phonon dispersion of monoclinic II (I2/m) phase. The imaginary mode at the Γ point is indicated by *u*_1_. In (c) we display the BiO_6_ rotations in anticlockwise (left) and clockwise (right) directions.

In **Figure** **6**a we show another aspect of the anharmonicity of BaBiO_3_. We find a double‐well potential energy associated with the rotation of BiO_6_ octahedra in the monoclinic II phase (I2/m). Similar double‐well potentials were early observed for the cubic Ba_0.5_K_0.5_BiO_3_ compound.^[^ ^82^ ^]^ In our case, the double‐well potential was obtained by considering the distortion of the monoclinic II phase along the normal mode coordinate $Q_{u_{1}}$, associated with the imaginary mode *u*_1_ at the Γ point shown in Figure 6b. In Figure 6a, the continuous line represents a 20th order polynomial fit of DFT total energies of distorted structures (red circles). A similar procedure was adopted in Ref. [83]. This fingerprint of anharmonicity is also significant in setting up the energy splitting of a ‘particle’ within the double‐well potential, which is of great importance for the resonant scattering of acoustic phonons.

In fact, by solving the 1D Schrödinger equation for an oxygen atom (m_O_ = 15.99 amu) within the double‐well potential^[^ ^84^ ^]^ shown in Figure 6a, we find an energy splitting of Δ_min_ ≈ 1.2 meV. Δ_min_ represents the energy difference between the ground and the first excited state. Hence, within a TTLS picture, we expect a strong resonant scattering of acoustic phonons around 14 K. More importantly, we find that Δ_min_ is in reasonable agreement with the thermal energy of the dominant phonon (*E* ∼ *k*_*B*_ *T*) computed at the temperature of the onset of the plateau (around 20 K) observed in our thermal conductivity measurements. The slight deviation between the simplified TTLS model and the experimental data is possibly related to the expected distribution of splitting energies due to strain fields, point defects, and possible interactions between TTLS.^[^ ^85^ ^]^ Upon increasing the temperature, the high energy states within the double‐well potential are excited up to the barrier height, which corresponds to Δ_max_ ≈ 31  meV, providing further resonant scattering frequencies for the phonons and contributing to the appearance of the plateau in the thermal conductivity. The maximum energy splitting Δ_max_ indicates that the TTLS may effectively scatter phonons up to 360 K, partially explaining why our plateau extends up to 260 K. Another important feature one can notice in our thermal conductivity data is the enhancement of phonon scattering at around 70 K (change of slope in the plateau), which corresponds to the energy of about 6 meV. This energy is close to energies of the lowest optical mode (≈5.1 meV), which can also scatter the acoustic phonons. Therefore, our findings suggest the existence of an interplay of a phonon scattering due to TTLS and low‐energy optical modes in intermediate temperatures. We emphasize that the inclusion of phonon scattering by two‐level tunneling systems (TTLS) in the theoretical approximations for calculating lattice thermal conductivity is beyond the scope of this work; however, it will be of crucial importance for a deeper understanding of glass‐like behaviors in thermal properties of high‐quality single‐crystals, not only in BaBiO_3_.

Further experiments will be fundamental to pin down the tunneling splitting energy and the microscopic origin of the tunneling states, which we believe rule the temperature evolution of the thermal conductivity of BaBiO_3_. We stress that a glass‐like thermal conductivity and experimental evidences of a TTLS were recently reported for single‐crystals of BaTiS_3_ in Ref. [8]. It is worth mentioning that the discrepancy between the theoretical and measured κ(*T*) in their case is similar to the one observed in our study. This observation indicates that relevant phonon scattering induced by TTLSs may be a more common feature among materials with sizable anharmonicity and dynamical structural instabilities. It is important to clarify if the same complex interplay takes place in halide perovskites, which can exhibit dynamic structural instabilities.^[^ ^80^ ^]^

## 7 Conclusion

Our work reports direct experimental evidence of an anomalous glassy thermal conductivity in single‐crystals of BaBiO_3_, with a (*i*) ∼*T* ^2^ dependence at low temperatures (*T* < 5 K), a (*ii*) plateau in a wide temperature range (20 K <*T* < 260 K), and a (*iii*) surprising recovery of a downturn for *T* > 300 K. The low magnitudes of thermal conductivity at high temperatures were well described by state‐of‐the‐art mode‐coupling theory calculations for the lattice thermal conductivity. We found that three‐phonon scattering, associated with the sizable acoustic‐optical coupling and implicit anharmonicity, leads to the suppression of κ, though, it cannot describe the suppression of κ(*T*) for *T* < 260 K, indicating the existence of additional scattering mechanisms. We propose that the presence of a TTLS, associated with the dynamic structural instability of BaBiO_3_, may be the missing source of additional phonon scattering.

The lattice anharmonicity of BaBiO_3_ may also impact the pairing of charge carriers due to incoherent phonons^[^ ^86^, ^87^ ^]^ and the emergence of a superconducting phase in the doped compound as well as in the insulating phase out‐of‐equilibrium.^[^ ^45^ ^]^ The observed very low thermal conductivity suggests BaBiO_3_ as a promising building block for functional heterostructures and superlattices, allowing for an effective filter of the heat carriers. Finally, our findings indicate that oxides with unusual structural instabilities associated with the oxygen octahedra are promising candidates for new low‐thermal conductivity materials.

## 8 Experimental and Computational Methods

The thermal conductivity of commercial BaBiO_3_ single crystals was investigated in the 1.5 – 300 K temperature range, using a standard two‐thermometers, one‐heater technique on a custom‐built setup. For 300 K <*T* < 400 K, a setup based on 3ω principle was employed.^[^ ^88^ ^]^ The details on the thermal conductivity measurements and their uncertainties were outlined in the Section S1 (Supporting Information). The structure and alignment of the samples were verified by neutron single crystal x‐ray diffraction (SXD) and Laue diffraction carried out at the ISIS Neutron and Muon Source (See Sections S2 and S3, Supporting Information). The crystals showed the expected monoclinic phase at room temperature. It must be highlighted that it did not detect any evidence of diffuse scattering during the neutron‐SXD experiments for a long time of beam exposure (> 20hrs), pointing to an undetectable level of static disorder and a very high quality of the crystals. The method used for measuring specific heat was described in the Section S4 (Supporting Information). The lattice dynamics and anharmonicity of BaBiO_3_ were studied by means of first principles calculations. Ab‐initio MD calculations were also carried out for the monoclinic II and cubic phases, to obtain the high‐order interatomic force constants (IFCs) at finite temperatures, within the temperature‐dependent effective potential technique (TDEP).^[^ ^23^, ^89^, ^90^, ^91^ ^]^ For the monoclinic II phase, the obtained IFCs was used to calculate of the lattice thermal conductivity within the mode‐coupling theory.^[^ ^28^, ^29^ ^]^ The computational details relative to these calculations are described in the Sections S5 to S10 (Supporting Information).

## Conflict of Interest

The authors declare no conflict of interest.

## Author Contributions

V.M. designed the research. A.H. and M.S.S.L. performed the thermal conductivity and specific heat experiments. A.H. performed SXD and Laue experiments in collaboration with G.N., M.J.G., and S.W. W.H.B. performed the DFT, MD, and lattice thermal conductivity calculations, and theoretical analysis. V.M., W.H.B., A.H., and M.S.L.L. wrote the manuscript and discussed the results with input from all other authors.

## Supporting information

Supporting Information

## Data Availability

The data that support the findings of this study are available in the supplementary material of this article.
