# Supplementary material for: Anomalous Glassy Thermal Conductivity in a Perovskite Bismuthate Induced by Structural Dynamic Instability
Source: Adv Sci (Weinh). 2025 Jun 4;12(31):e02379. doi: 10.1002/advs.202502379 (PMC12376643; doi:10.1002/advs.202502379)
Supplement: Supplementary file 1 — Supporting Information [file ADVS-12-e02379-s001.pdf]

ADVANCED SCIENCE
Open Access

Supporting Information

for *Adv. Sci.*, DOI 10.1002/advs.202502379

Anomalous Glassy Thermal Conductivity in a Perovskite Bismuthate Induced by Structural Dynamic Instability

Alexandre Henriques, Mariana S. L. Lima, Gøran J. Nilsen, Matthias J. Gutmann, Steffen Wirth, Walber H. Brito and Valentina Martelli**

Anomalous glassy thermal conductivity in a perovskite bismuthate induced by structural dynamic instability: Supplemental Material

Alexandre Henriques[1], Mariana S. L. Lima[1], Gøran J. Nilsen[2], Matthias J. Gutmann[2], Steffen Wirth[3], Walber H. Brito[4,5*], and Valentina Martelli[1,*]
(1) Institute of Physics, University of São Paulo, 05508-090, São Paulo, Brazil
(2) ISIS Neutron and Muon Source, Didcot, Oxfordshire, OX11 0QX,UK
(3) Max Planck Institute for Chemical Physics of Solids, D-01187 Dresden, Germany
(4) Department of Physics, Federal University of Minas Gerais, Belo Horizonte, 31270-901, Brazil
(5) Department of Physics and Astronomy, Rutgers University, Piscataway, New Jersey 08854, USA

S1. Thermal conductivity of $BaBiO_3$: methods

Stationary method. Thermal conductivity $\kappa(T)$ was measured through the standard one-heater-two-thermometers method in a custom-built platform attached to the low-temperature stage of a cryostat operating in the 2-300 K range. The two temperature sensors (Cernox-1050) were first calibrated in the aforementioned temperature range by interpolating an 8th order Chebyshev polynomial to the resistance data. The longitudinal thermal conductivity is computed as $\kappa = P/(g\Delta T)$. P is the power provided by a resistor heater that establishes a temperature gradient between the two heaters, ΔT, throughout a specimen of geometrical factor $g = A/\ell$ (the ratio of the cross-section area and the average distance between the thermometers). When the gradient was established, the temperature at which the thermal conductivity is determined, $\tilde{T}$, was taken as the average of the two sensors. The thermal gradient is established with a 1.5 kΩ resistor fed by a DC current Lakeshore 155 V/I Source, while the temperatures of the sensors were monitored using a Lakeshore 372 AC Resistance Bridge. We actively kept the ratio $\Delta T/\tilde{T}$ close to 1%, besides keeping the temperature of the platform stable through active PID control. The main source of uncertainty in the values of thermal conductivity comes from the determination of the geometrical factor, and the total relative uncertainty σ_κ/κ is about 9%.

3ω method. Thermal conductivity of single-crystalline $BaBiO_3$ was also measured through the 3ω technique [1] up to 395 K. Measurements above room temperature were carried out in a custom-built vacuum chamber with a water-cooled cold finger to guarantee heat dissipation. At each temperature setpoint, a third harmonic frequency sweep is performed, $W_{3\omega}$, as shown in Figure S1 for selected temperatures. The stability of the platform's base temperature T_∞ is monitored by a Cernox-1050 temperature sensor along all frequency scans. A 100 nm-thick Pt metal line was evaporated by shadow-masking a "H"-shaped geometry with four contact pads, over a 0.5 mm-thick single crystal $BaBiO_3$ specimen, and acts as a heater/thermometer (H/T) transducer [2]. The metal line full-width was patterned as $2b_h = 100$ μm and length ℓ_h =1.10 mm.

For the cancellation of the first harmonic signal, we have employed a custom built Wheatstone Bridge and digital resistance decade PRS 330 by IET labs and the detection of the third harmonic signal, $W_{3\omega} \propto V_{3\omega}$, and monitoring of the constant first harmonic voltage are accomplished using two 7270 General Purpose DSP Lock-in Amplifier manufactured by AMETEK scientific instruments. The thermal conductivity at the corresponding temperature is obtained after data analysis with the 3ω method formalism. Technical details of our implemented setup can be found in Ref.[2]. Measurements were performed inside a custom-built high-temperature vacuum chamber from 300 - 395 K. The pressure inside the experimental sample space was $< 6.0 \times 10^{-4}$ mbar. The driving power per unit length for the measurements was kept close to 1.9 W/m. The main source of uncertainty in the values of thermal conductivity via the 3ω method comes from the H/T calibration curve [2], and the total relative uncertainty σ_κ/κ is about 12%.

Given the working principle of the two techniques and the shape of the samples determined by the growth (thin parallelepipeds) $\kappa(T)$, determined by the two techniques, probes two orthogonal directions in the crystals. $\kappa(T)$ measured by the standard one-heater/two-thermometer setup probes thermal transport perpendicular to the (100) crystallographic direction, while $\kappa(T)$ determined by 3ω is along the (100)-direction. We observe a mismatch at room temperature of 1.6 - 1.8 W/mK. However, we cannot firmly ascribe it to anisotropy as the difference, in the order of 10%, is comparable to the error bars.

S2. SXD neutron scattering refinement

Room temperature SXD experiments were conducted at the ISIS Neutron and Muon Source (Rutherford Appleton Laboratory) facility's dedicated single-crystal diffractometer SXD [3]. We investigated commercial $BaBiO_3$ crystals

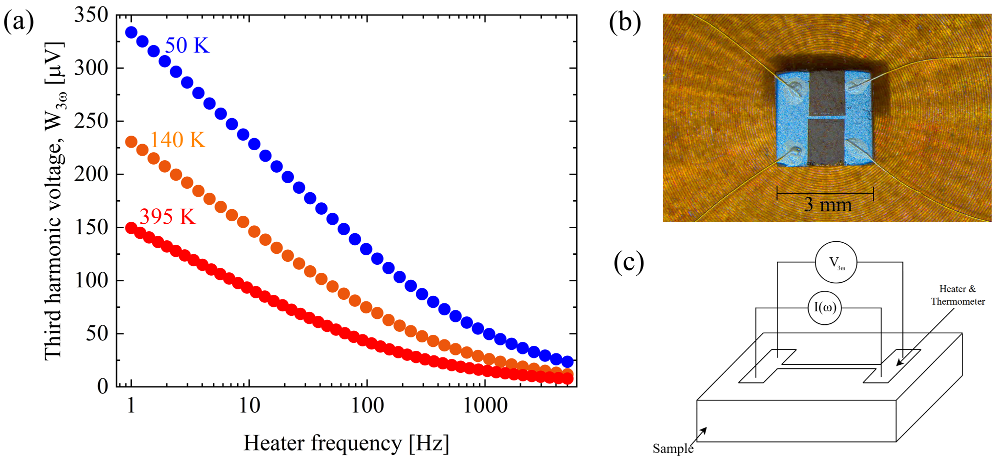

FIG. S1: (a) Third harmonic voltage sweeps as a function of frequency at fixed temperatures T_∞. The slope of the voltage as a function of the input excitation current frequency is inversely proportional to the thermal conductivity of the sample [1]. (b) $BaBiO_3$ crystal with Pt-deposited electrode (H/T) for a 3ω thermal conductivity experiment and (c) scheme of electrical contacts, both for current input $I(\omega)$ and measurement of the third harmonic voltage $W_{3\omega} \propto V_{3\omega}$.

(100) oriented with alignment tolerance of 0.3°, grown by SurfaceNet GmbH. Crystal dimensions were about $2 \times 2 \times 0.5$ mm^3, with the (100) direction being perpendicular to the 2×2 mm^2 surface. In particular, SXD can resolve twinned domains in terms of volume fraction and twinning, which is of great interest in the present case of $BaBiO_3$. Due to the different element sensitivities, neutron diffraction data provide complementary information to high resolution X-ray studies. Each neutron beam exposition (at fixed temperature) took at least 10h, for improved statistics. The data analysis and single-crystal refinements are done using the Jana2006 program [4], and the summary of the refinements is shown in Table S1.

TABLE S1: Information sheet from single crystal neutron diffraction.

	**Compound**	**$BaBiO_3$**			
	Refined formula	$Ba_2Bi^{3+}Bi^{5+}O_6$			
	Space group	I2/m (12)			
	Structural phase	Monoclinic II			
	Formula weight	394.31 g/mol			
	R_{wp}	8.96 %			
	Atomic coordinates Å and equivalent atomic displacement parameters /Å				
	x	y	z	Occ	U(eq)
Ba	0.49973	0.00000	0.25220	1.000	0.006
Bi^{3+}	0.00000	0.00000	0.00000	1.000	0.006
Bi^{5+}	0.00000	0.00000	0.50000	1.000	0.003
O_1	0.05941	0.00000	0.26078	1.000	0.021
O_2	0.26293	0.25687	-0.03295	1.000	0.023

S3. Laue diffraction

By transverse Laue X-rays scans over the entire sample surface, using evenly spaced steps of 0.5 mm, we found no irregularity of the diffraction spots - see Fig. S2. Data were collected at room temperature. The intense brightness of the spots indicates high-quality growth. Notice that the typical beam size is about 200 μm, therefore the coherence of the crystalline domains extends beyond the illuminated area. Some degree of twinning is present, as one can observe

two different high-symmetry patterns embedded in the picture. Through a simulation done with QLAUE software (version 0.2), we have identified a crystallographic orientation corresponding to (100), compatible with the direction indexed by the manufacturer (SurfaceNet). Extra spots belong to a different orientation induced by crystallographic twinning. The monoclinic (M) crystal structure is known to allow for twinned domains, as a monoclinic unit cells may have a higher symmetry than implied by the space group, specially in situations where $\beta \sim 90°$ [5], which is precisely the present case. This is not the first time twinned domains were reported in $BaBiO_3$ - see for instance Foyevtsov and co-workers [6] Such domains are formed post crystal growth, because $BaBiO_3$ grows in a cubic (C) structure above $\sim$ 800 K, where no twinning is possible. Upon cooling, intermolecular interactions seem to be not so energetically favorable in comparison to what would be formed in a single crystal, allowing for a considerable twin fraction.

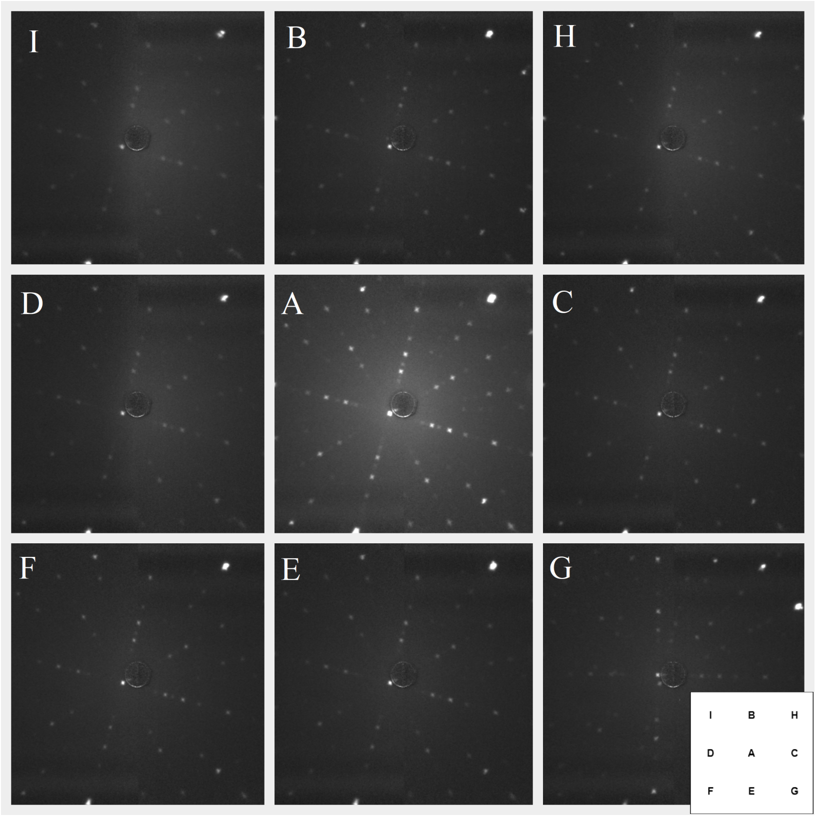

FIG. S2: Laue patterns of different spots over the sample's surface. Each letter indicates the relative position of the measurements, separated by 0.5 mm. The clear consistency points to a high homogeneity of the crystals. The central picture (A) shows brighter intensities due to longer exposition time (3 minutes).

S4. Specific heat measurements and analysis

Specific heat $c(T)$ was measured between 0.25 K and 400 K using a standard platform in a PPMS Quantum Design cryogenic system with a DynaCool Dilution Refrigerator. In this Supplementary material, we report the details of the $c(T)$ analysis and estimate the Debye temperature.

The Debye term describes the lattice contribution to the heat capacity, and is given by the formula (per atom)

$$C_{\mathrm{D}}(T) = 3R\left(\frac{T}{\Theta_D}\right)^3 \int_0^{\Theta_D/T} \frac{x^4 e^x}{(e^x-1)^2}dx \tag{S1}$$

where $x = \hbar\omega/k_BT$. The Einstein equation for specific heat is

$$C_{\mathrm{E}}(T) = R\frac{e^{\Theta_E/T}}{(e^{\Theta_E/T}-1)^2}\left(\frac{\Theta_E}{T}\right)^2 \tag{S2}$$

At very low temperatures, $\Theta_D/T \gtrsim 50$, Equation (S1) can be approximated to $c = \beta T^3$, where

$$\beta = \frac{12\pi^4}{5}\frac{N_{\rm ions}N_A}{\Theta_D^3} \tag{S3}$$

Hence, the Debye temperature can be determined through

$$\Theta_D = \left(\frac{12\pi^4}{5}\frac{N_{\rm ions}N_A k_B}{\beta}\right)^{1/3} \tag{S4}$$

where $N_{\rm ions} = 5$ is the number of atoms (ions) in the unit cell, N_A is Avogadro's number, and k_b is the Boltzmann constant. Herein, we have found $\Theta_D = 410 \pm 12$ K. This value is larger than that observed by Kuentzler et al. ($\Theta_{Debye} = 230$ K) [7]. Note that this reference has not mentioned the need for extra terms (i.e. Einstein modes) in the model of specific heat.

Alternatively, we used the standard method to calculate the Debye temperature from data of elastic constants, since Θ_D is proportional to the average sound velocity $v_{\rm avg}$ [8, 9] :

$$\Theta_D = \frac{h}{k_B}\left[\frac{3}{4\pi}\frac{N_A\rho}{M}\right]^{1/3} v_{\rm avg} \tag{S5}$$

where h is Planck's constant, k_B is Boltzmann's constant, ρ is the volumetric density, and M is the molecular weight of the solid. $v_{\rm avg}$ was determined by the Voight-Reuss-Hill (VRH) approximation as discussed in Section S5.

We firstly fit the low temperature range using $c = \gamma_{\rm TS}T^{1.3} + \beta T^3$ with the convention $\Theta_D/T < 50$ [7], where the first term corresponds to the specific heat of tunneling states (TSs) and the second term represents the approximation of lattice heat capacity (Debye) at low temperatures. Doing so allows to remove the TSs contribution to the total specific heat data ($\gamma_{\rm TS} = 4.490\times10^{-4}$ J mol^{-1} K$^{-2.3}$), and the Debye temperature can be estimated as follows.

From the best-fit value of $\beta = 3.623 \times 10^{-4}$ J mol^{-1} K^{-4} , and using Equation (S5), we determined $\Theta_D = 410 \pm 12$ K. Since the average sound velocity was found to be 2772 m/s (See Section S6), from which we determined $\Theta_D^{\rm calc} = 413$ K, in reasonable agreement with the experimental Debye temperature. We wish to point out that in Ref. [7] a factor $\sqrt[3]{5}$ is missing that comes from not accounting for the number of ions in the unit cell ($N = 5$). Moreover, at high temperatures, *c(T)* approaches 120 J mol^{-1}K^{-1}, consistent with the expected Dulong-Petit value of 3NR = 124 J mol^{-1}K^{-1} for a solid with $N = 5$ atoms in the unit cell.

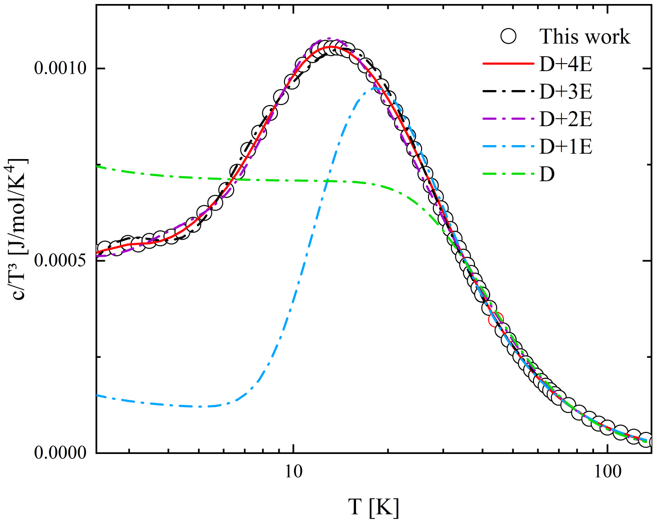

FIG. S3: **Specific heat data and D + NE model fits.**

The model used to fit the specific heat data in the temperature range 2 K - 150 K is the standard Debye model with the contribution of the Einstein model with characteristic temperature Θ_E (see for instance [10]) as follows:

$$C(T) = C_{\rm D}\left(T, \Theta_D\right) + \sum_{i=1}^{4} r_{\rm E,i} C_{\rm E}(T, \Theta_{\rm E,i}) \tag{S6}$$

where Θ_D is the Debye temperature, Θ_E is the Einstein temperature with respective weights $r_{\rm E,i}$. The relative weight of the Debye term, $r_{\rm D}$, has been omitted throughout the text for simplicity.

We have found the best modeling of our data after progressively increasing the number of Einstein terms and evaluating the goodness-of-fit, until four terms attained to the R-squared closer to unit, as shown in Figure S3 and Table S2. The obtained Debye temperature $\Theta_D = 424$ K is in agreement with what was found above by the low temperature approximation - Equation (S5).

TABLE S2: Progressive improvement of the D+NE model by the inclusion of more Einstein terms, until optimal convergence with N = 4 terms.

Θ_D [K]	$\Theta_{\rm E,1}$ [K]	$\Theta_{\rm E,2}$ [K]	$\Theta_{\rm E,3}$ [K]	$\Theta_{\rm E,4}$ [K]	R-squared
202	-	-	-	-	0.1377
372	90	-	-	-	0.7175
227	27	66	-	-	0.9685
317	15	45	93	-	0.9933
424	13	34	67	128	0.9993

S5. Computational methods

DFT, lattice dynamics, and MD. To obtain the phonon dispersions and Grüneisen parameters, we performed density functional theory (DFT) calculations within the Perdew-Burke-Ernzehof generalized gradient approximation [11], using projector augmented wave (PAW) potentials [12], as implemented in the Vienna Ab initio Simulation Package (VASP) [13, 14]. A plane wave cut-off of 550 eV was used and total energies were converged within 1×10^{-8} eV criterion. A k-point mesh of $4 \times 4 \times 4$ was used to obtain the total energies and interatomic forces. The phonon dispersion of the monoclinic I ($P2_1/n$) phase was obtained within the supercell-based frozen phonon approximation, with displacements generated using $2\times2\times2$ supercells. The second-order interatomic force constants (IFC) were obtained, using these $2\times2\times2$ supercells, employing VASP and PHONOPY [15, 16]. A similar procedure were adopted for the phonon dispersion of the monoclinic II phase, which is shown in Fig. 6(b) of the main text. To obtain the spectral function of the cubic phase we performed MD simulation using 3000 steps (using time steps of 1 fs) within the canonical NVT ensemble, considering a $2 \times 2 \times 2$ supercell. For the monoclinic II phase we also performed molecular dynamics for $T = 300$ K using a $2 \times 2 \times 2$ supercell. In this case, we obtained on-the-fly machine learning force fields [17–19] within the NPT ensemble for 8 ps. Using the machine learning force fields, additional MD calculations were performed (using a $4 \times 4 \times 4$ supercell, 540 atoms) for 30 ps.

TDEP. The second and third order force constants were extracted for the cubic phase using the MD calculations and the temperature-dependent effective potential technique (TDEP) [20–23]. The same procedure was used for the monoclinic II phase, where we obtained the second, third and fourth order IFCs. We emphasize that within TDEP method, the anharmonicity is introduced by fitting an effective Hamiltonian, at each temperature, using the results obtained with MD. Within this theoretical framework, phonon line-shapes and spectral functions can be calculated. The phonon spectral function obtained for the cubic phase (at 850 K) is shown in Fig. S4.

S6. Sound Velocity

We recall the stress-strain relation in an orthotropic monoclinic crystal can be defined by the independent elastic stiffness parameters using the Voigt-Reuss-Hill (VRH) approximation [24–26] :

$$\begin{bmatrix} \sigma_{11} \\ \sigma_{22} \\ \sigma_{33} \\ \sigma_{12} \\ \sigma_{13} \\ \sigma_{23} \end{bmatrix} = \begin{bmatrix} C_{11} & C_{12} & C_{13} & 0 & C_{15} & 0 \\ C_{12} & C_{22} & C_{23} & 0 & C_{25} & 0 \\ C_{13} & C_{23} & C_{33} & 0 & C_{35} & 0 \\ 0 & 0 & 0 & C_{44} & 0 & C_{46} \\ C_{15} & C_{25} & C_{35} & 0 & C_{55} & 0 \\ 0 & 0 & 0 & C_{46} & 0 & C_{66} \end{bmatrix} \begin{bmatrix} \varepsilon_{11} \\ \varepsilon_{22} \\ \varepsilon_{33} \\ \gamma_{12} \\ \gamma_{13} \\ \gamma_{23} \end{bmatrix} \tag{S7}$$

where C_{ij} are the components of the elastic constants tensor, σ represents the normal and shear stress, ε, and γ are normal and shear strain in each direction. The calculated elastic constants within the energy-strain method [27] is listed in Table S3. We also list the results obtained in Ref. [28] for comparison.

TABLE S3: Calculated elastic constants C_{ij} (in GPa) of $BaBiO_3$ for monoclinic I. Calculations from Gao et al. (2020) [28] are indexed for comparison.

C_{ij}	Gao et al. (2020) [28]	This work
C_{11}	202.2	172.1
C_{22}	178.6	163.5
C_{33}	191.5	176.5
C_{44}	53.2	41.8
C_{55}	49.0	44.5
C_{66}	69.1	50.5
C_{12}	78.3	83.9
C_{13}	66.0	83.9
C_{15}	-3.8	2.0
C_{23}	54.8	53.9
C_{25}	-3.5	-4.6
C_{35}	5.0	3.1
C_{46}	-3.7	-2.4

For monoclinic crystal structures, the relation between the elastic constants and bulk modulus (B) and shear modulus (G) in the VRH approximation is given by [8, 24–26]:

$$B = \frac{(B_V + B_R)}{2} \tag{S8}$$

$$G = \frac{(G_V + G_R)}{2} \tag{S9}$$

$$\tag{S10}$$

The B_V and G_V are the maximum moduli for monoclinic symmetry :

$$B_V = [C_{11} + C_{22} + C_{33} + 2(C_{12} + C_{13} + C_{23})]/9 \tag{S11}$$

$$G_V = [C_{11} + C_{22} + C_{33} - C_{12}C_{13} - C_{23} + 3(C_{44} + C_{55} + C_{66})]/15 \tag{S12}$$

The B_R and G_R are minimum moduli for monoclinic symmetry:

$$\begin{aligned} B_R &= \Omega/[(a(C_{11} + C_{22} - 2C_{12}) \\ &+ b(2C_{12} - 2C_{11} - C_{23}) \\ &+ c(C_{15} - 2C_{25}) \\ &+ d(C_{12} + 2C_{23} - C_{13} - 2C_{22}) \end{aligned}$$

$$
\begin{aligned}
G_R &= 15/[(4(a(C_{11}+C_{22}+C_{12})+b(C_{11}-C_{12}-C_{23})\\
&+ c(C_{15}+C_{25})+d(C_{22}-C_{23}-C_{12}-C_{13})\\
&+ e(C_{25}-C_{15})+f))\Omega\\
&+ 3(g\Omega+C_{44}+C_{66})/(C_{44}C_{12}-C_{46}C_{46})
\end{aligned}
$$

where

$$a = C_{33}C_{55} - C_{35}^2,$$

$$b = C_{23}C_{55} - C_{25}C_{35},$$

$$c = C_{13}C_{35} - C_{15}C_{33},$$

$$d = C_{13}C_{55} - C_{15}C_{35},$$

$$e = C_{13}C_{25} - C_{15}C_{23},$$

$$f = (C_{11}(C_{22}C_{55} - C_{25}^2)) - (C_{12}(C_{12}C_{55} - C_{15}C_{25})) + \\ (C_{15}(C_{12}C_{25} - C_{15}C_{22})) + (C_{25}(C_{23}C_{35} - C_{25}C_{33}))$$

$$g = C_{11}C_{22}C_{33} - C_{11}C_{23}C_{23} - \\ C_{22}C_{13}C_{13} - C_{33}C_{12}C_{12} + 2C_{13}C_{12}C_{23}$$

$$\Omega_1 = (C_{15}C_{25}(C_{33}C_{12} - C_{13}C_{23})) + \\ (C_{15}C_{35}(C_{22}C_{13} - C_{12}C_{23})) + \\ (C_{25}C_{35}(C_{11}C_{23} - C_{12}C_{13}))$$

$$\Omega_2 = (C_{15}^2(C_{22}C_{33} - C_{23}^2)) + \\ (C_{25}^2(C_{11}C_{33} - C_{13}^2)) + \\ (C_{35}^2(C_{11}C_{22} - C_{12}^2))$$

$$\Omega = 2\Omega_1 - \Omega_2 + gC_{55}$$

The criteria for mechanical stability are [24–26] :

$$C_{ij} > 0 (i = j, 1, 2, 3, 4, 5, 6) \quad \text{(S13)}$$

$$(C_{44}C_{66} - C_{46}^2) > 0 \quad \text{(S14)}$$

$$(C_{33}C_{55} - C_{35}^2) > 0 \quad \text{(S15)}$$

$$(C_{22} + C_{33} - 2C_{23}) > 0 \quad \text{(S16)}$$

$$[C_{11} + C_{22} + C_{33} + 2(C_{12} + C_{13} + C_{23}) > 0 \quad \text{(S17)}$$

$$\Omega > 0 \quad \text{(S18)}$$

Young's modulus (Y) and the Poisson ratio (ν) can be used to evaluate the stability of materials. They are calculated from B and G [24, 26]:

$$Y = \frac{9BG}{3B + G} \quad \text{(S19)}$$

$$\nu = \frac{3B - 2G}{2(3B + G)} \quad \text{(S20)}$$

(S21)

Furthermore, the transverse acoustic velocity $v_{\rm T}$, longitudinal acoustic velocity $v_{\rm L}$, average sound velocity $v_{\rm avg}$ are calculated as [8]:

$$v_{\rm L} = \sqrt{\frac{G}{\rho}} \tag{S22}$$

$$v_{\rm T} = \sqrt{\frac{B + \frac{4G}{3}}{\rho}} \tag{S23}$$

$$v_{\rm avg} = \left[\frac{1}{3}\left(\frac{2}{v_{\rm T}^3} + \frac{1}{v_{\rm L}^3}\right)\right]^{-1/3} \tag{S24}$$

In table S4 we display the obtained values of B, G, Y, ν, and velocities for $BaBiO_3$ (monoclinic I phase).

TABLE S4: Calculated bulk modulus (B), shear modulus (G), Young's modulus (Y) and the Poisson ratio (ν), the transverse acoustic velocity $v_{\rm T}$, longitudinal acoustic velocity $v_{\rm L}$, average sound velocity $v_{\rm avg}$ of $BaBiO_3$ (monoclinic I phase). Calculations from Gao et al. (2020) [28] are also indexed for comparison.

Parameters	Gao et al. (2020) [28]	This work
B (GPa)	107.5	103.4
G (GPa)	58.4	46.9
Y (GPa)	148.3	122.2
ν	0.27	0.30
$v_{\rm T}$ (m/s)	2731	2481
$v_{\rm L}$ (m/s)	4865	4667
$v_{\rm avg}$ (m/s)	3039	2772

S7. Average frequencies and spring constant of atoms in $BaBiO_3$

As discussed by Feng and co-workers [29], assigning the most weakly bound atom may be achieved by semi-classically treating the bonds and atoms as spring-mass systems. Assuming that the atoms oscillate in an average frequency, $\langle f\rangle_\alpha$, the spring constant associated with the atom of mass m_α is $k_\alpha = m_\alpha\langle\omega\rangle^2_\alpha$, where $\omega_\alpha = 2\pi f_\alpha$. The averaged frequency is calculated as the "center of mass" of the projected phonon density of states (PPDOS) for each atom α (shown in Fig.2(b) of the main text),

$$\langle f_\alpha\rangle = \frac{\int_0^\infty \nu \mathrm{PPDOS}_\alpha(\nu)d\nu}{\int_0^\infty \mathrm{PPDOS}_\alpha(\nu)d\nu}. \tag{S25}$$

Table S5 shows the calculated averaged frequencies and the corresponding spring constants of the constituent atoms of $BaBiO_3$. For computing the average frequencies, we used the monoclinic I ($P2_1/n$) symmetry; we have found a slight change of about 2% by using the monoclinic II symmetry (I2/m).

One can notice that the barium atoms present a relatively small spring constant, leading to $k_{\rm min}/k_{\rm max} = \langle k\rangle_{\rm Ba}/\langle k\rangle_{\rm Bi2} = 0.246$. The concentration of Ba modes close to $\sim$ 70 cm^{-1} as shown in Fig. 2(b) (main text) justifies such a relatively low average frequency. Feng et al. (2020) [29] have investigated a handful of half-Heusler compounds (chemical formula ABC, regarded as filled zinc blende materials) in terms of this metric. They showed that there is a clear correlation between low $k_{\rm min}/k_{\rm max}$ ratio, low room temperature thermal conductivity (≤ 2 W m^{-1} K^{-1}) and sizable anharmonicity (manifested as rattler atoms especially in PtLaSb, RhLaTe, BiBaK, and PCdNa). We point out that $BaBiO_3$ presents similar values of thermal conductivity, Debye temperature and average frequency to those four mentioned compounds, hence suggesting that indeed weak Ba-O bonds lead to phonon-scattering mechanisms broadly regarded to as rattling.

TABLE S5: Average frequencies and spring constants of Ba, Bi and O atoms in $BaBiO_3$. We distinguish between the two inequivalent Bi-sites. Within the charge-disproportionation (CD) picture, Bi1 and Bi2 are labeled as Bi^{3+} and Bi^{5+}, respectively.

Atom	$\langle f \rangle_\alpha$ [THz]	$\langle \nu \rangle_\alpha$[cm^{-1}]	$\langle k \rangle_\alpha$ [kg/s^2]
Ba	2.64	88	63
Bi1	3.19	106	140
Bi2	4.32	144	256
O	8.32	277	73

S8. Phonon spectral function of cubic phase

The obtained phonon spectral function, at 850 K, for the cubic $BaBiO_3$ is shown in Fig. S4. We observe avoided crossing points along M-Γ and Γ-R high-symmetry directions.

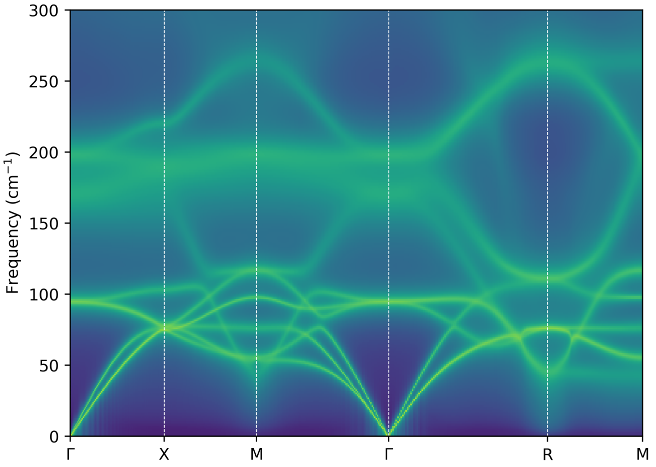

FIG. S4: Phonon spectral function of cubic $BaBiO_3$ phase obtained at 850 K.

S9. Thermodynamic properties of $BaBiO_3$ (monoclinic - $P2_1/n$) within QHA

To calculate the specific heat of the monoclinic I phase we performed DFT calculations for five different volumes. The convergence of calculations were also crosschecked using seven different volumes. Within the quasi-harmonic approximation (QHA), the phonon contribution to the Helmholtz Free energy is given by [30],

$$F_{ph}(V,T) = \frac{1}{2}\sum_{\mathbf{q}s} \hbar\omega_s(\mathbf{q},V) + k_BT\sum_{\mathbf{q}s} \ln\left[1 - \exp\Big(-\frac{\hbar\omega_s(\mathbf{q},V)}{k_BT}\Big)\right], \tag{S26}$$

where $\omega_s(\mathbf{q},V)$ is the phonon frequency with wave vector $\mathbf{q}$ and branch index s at volume V. The obtained values of the Free energy $F(V,T) = U(V) + F_{ph}(V,T)$ for different volumes are temperatures are shown in Fig. S5(a). $U(V)$ is the total energy of the electronic structure at constant volume. By minimizing the Free energy $F(V,T)$ with respect to the volume V at each temperature, we obtain $V(T)$ shown in Fig. S5(b). The bulk modulus (shown in Fig. S5(c)) is obtained using the Vinet equation of state (EOS).

S10. Theoretical approximation for lattice thermal conductivity of $BaBiO_3$

We calculated the lattice thermal conductivity of $BaBiO_3$ within the mode-coupling theory [31, 32]. In this theory, the thermal conductivity tensor is expressed as $\kappa = \kappa^d + \kappa^{od}$. The diagonal component κ^d of thermal conductivity

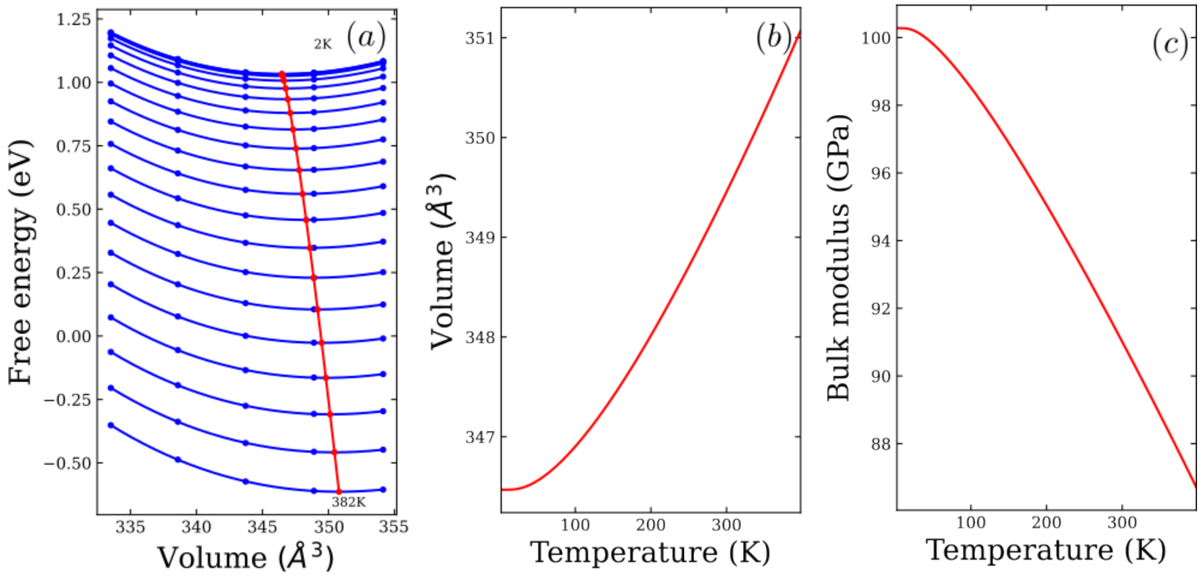

FIG. S5: (a) Free energy, (b) volume, and (c) bulk modulus of $BaBiO_3$ obtained within the QHA. In (a) we obtained the Free energy from 2 K up to 382 K ($\Delta T = 2$ K).

can be written as [32]

$$\kappa^d = \frac{1}{V} \sum_{\mathbf{q}_1\mathbf{q}_2} \sum_{s_1 s_2} \mathbf{v}_{\mathbf{q}_1}^{s_1} \otimes \mathbf{v}_{\mathbf{q}_2}^{s_2} c_{s_1}(\mathbf{q}_1)(\Xi^{-1})(\mathbf{q}_1 s_1, \mathbf{q}_2 s_2), \tag{S27}$$

where $\mathbf{q}$ denotes the wave vector, and s is the branch index for the mode $(\mathbf{q}, s)$. V is the system volume, $\mathbf{v}_{\mathbf{q}}^s$ is the phonon group velocity, $c_s(\mathbf{q})$ the modal heat capacity, and Ξ is the scattering matrix.

In an anharmonic crystal at finite temperature T, the renormalized phonon frequencies are denoted as $\Omega_s(\mathbf{q})$. In this case, the mode heat capacity is written as $c_s(\mathbf{q}) = \Omega_s^2(\mathbf{q}) n_s(\Omega_s(\mathbf{q}))[n_s(\Omega_s(\mathbf{q})) + 1]/k_B T^2$. Moreover, for interacting phonons, one can write a momentum and frequency-dependent phonon self-energy, $\Sigma_s(\mathbf{q}, \omega) = \Delta_s(\mathbf{q}, \omega) + i\Gamma_s(\mathbf{q}, \omega)$. Up to third order, the imaginary part can be expressed as $\Gamma_s(\mathbf{q}, \omega) = \Gamma_s^{(3)}(\mathbf{q}, \omega)$, which accounts for contributions arising from third-order interatomic force constants (IFCs), *i.e.*, three-phonon scattering processes. This term can be written as

$$\Gamma_s^{(3)}(\mathbf{q}, \omega) = \sum_{\mathbf{q}_2\mathbf{q}_3} \sum_{s_2 s_3} \Gamma_{s s_2 s_3}^{(3)}(\mathbf{q}, \mathbf{q}_2, \mathbf{q}_3, \omega), \tag{S28}$$

where the sum is over allowed energy and momentum conserving three phonon processes (normal and Umklapp), *i.e.* $\mathbf{q} + \mathbf{q}_2 + \mathbf{q}_3 = \mathbf{G}$, where $\mathbf{G}$ is a reciprocal lattice vector. Furthermore,

$$\Gamma_{s s_2 s_3}^{(3)}(\mathbf{q}, \mathbf{q}_2, \mathbf{q}_3, \omega) = \frac{\pi}{16} |\Psi_{\mathbf{q}\mathbf{q}_2\mathbf{q}_3}^{s s_1 s_2}|^2 \mathcal{S}^{(3)}(\omega, \Omega_{s_2}(\mathbf{q}_2), \Omega_{s_3}(\mathbf{q}_3)), \tag{S29}$$

$$\mathcal{S}^{(3)}(\omega, \Omega_{s_2}(\mathbf{q}_2), \Omega_{s_3}(\mathbf{q}_3)) = \sum_{s=1,-1} \{s[n(\Omega_{s_2}) + n(\Omega_{s_3}) + 1]\delta(\omega + s\Omega_{s_2} + s\Omega_{s_3}) + s[n(\Omega_{s_2}) - n(\Omega_{s_3})]\delta(\omega + s\Omega_{s_2} - s\Omega_{s_3})\}, \tag{S30}$$

where $n(\Omega_s(\mathbf{q})) = (\exp(\hbar\Omega_s(\mathbf{q})/k_B T) - 1)^{-1}$ is the Bose-Einstein distribution of phonon $(\mathbf{q}, s)$. From Γ_s^3, one can obtain the real part of the phonon self-energy using a Kramers-Kronig transformation [33],

$$\Delta_s^{(3)}(\mathbf{q}, \omega) = \frac{1}{\pi} \int \frac{\Gamma_s^{(3)}(\mathbf{q}, \Omega)}{\Omega - \omega} d\Omega. \tag{S31}$$

The matrix elements $\Psi^{ss_1s_2}_{\mathbf{q}\mathbf{q}_2\mathbf{q}_3}$ are the reciprocal-space representation of the third-order IFCs ($\Psi^{\alpha\beta\gamma}_{ijk}(\mu,\nu)$),

$$\Psi^{ss_2s_3}_{\mathbf{q},\mathbf{q}_2,\mathbf{q}_3} = \sum_{ijk}\sum_{\alpha\beta\gamma}\sum_{\mu\nu}\Psi^{\alpha\beta\gamma}_{ijk}(\mu,\nu)\frac{\epsilon^{i\alpha}_{s}(\mathbf{q})\epsilon^{j\beta}_{s_2}(\mathbf{q}_2)\epsilon^{k\gamma}_{s_3}(\mathbf{q}_3)}{\sqrt{M_iM_jM_k}\sqrt{\Omega_s(\mathbf{q})\Omega_{s_2}(\mathbf{q}_2)\Omega_{s_3}(\mathbf{q}_3)}}e^{-i(\mathbf{q}_2\mathbf{R}_{\mu,j}+\mathbf{q}_3\mathbf{R}_{\nu,k})}\Theta(\mathbf{q}+\mathbf{q}_2+\mathbf{q}_3), \tag{S32}$$

where i, j, and k are atoms in the unit cell and μ and ν denote the index of a unit cell in the crystal. In addition, $\mathbf{R}_{\mu,j}$ is the distance between the atom i in a reference unit cell and the atom j in the unit cell μ. $\Theta(\mathbf{q}') = 1$ if $\mathbf{q}' = \mathbf{G}$ and 0 otherwise. In the equation above, $\epsilon^{i\alpha}_{s}(\mathbf{q})$ is the displacement of atom i in cartesian direction α from the eigenvector of phonon mode $(\mathbf{q}, s)$, whereas M_i is the mass of atom i.

Another common source of phonon scattering is the presence of isotopic disorder, arising from the distribution of isotopes in the material. The imaginary part of the self-energy associated with this type of scattering can be expressed as

$$\Gamma^{iso}_s(\mathbf{q},\omega) = \sum_{\mathbf{q}_2s_2}\Gamma^{iso}_{ss_2}(\mathbf{q},\mathbf{q}_2,\omega) = \sum_{\mathbf{q}_2s_2}\sum_{i} g_i|\epsilon^i_s(\mathbf{q})\epsilon^i_{s_2}(\mathbf{q}_2)|^2\delta(\omega-\Omega_{s_2}(\mathbf{q}_2)), \tag{S33}$$

where $g_i = \sum_n \frac{d_{i,n}}{N}\left(\frac{\Delta M_{i,n}}{M_i}\right)^2$ according to Tamura's model [34]. g_i corresponds to the distribution of the isotope masses of element i, N is the number of isotopes, $d_{i,n}$ is the concentration of isotope n of element i, and $\Delta M_{i,n}$ is the mass difference between isotope n and the average mass of the element. In our case we used a natural distribution of isotopes. Therefore, in our calculations of the lattice thermal conductivity we considered $\Gamma_s(\mathbf{q},\omega) = \Gamma^{(3)}_s(\mathbf{q},\omega) + \Gamma^{iso}_s(\mathbf{q},\omega)$, *i.e.*, three-phonon and isotope scatterings. A more detailed discussion about these types of scattering can be found in Ref. [35].

Considering the phonon scattering mechanisms described above, the scattering matrix for the diagonal part of the thermal conductivity tensor can be written as follows [32],

$$\begin{aligned}\Xi_{s_1s_2}(\mathbf{q}_1,\mathbf{q}_2) = {} & \Gamma^M_{s_1}(\mathbf{q}_1)\delta_{\mathbf{q}_1\mathbf{q}_2}\delta_{s_1s_2}+ \\ & \frac{\Omega_{s_2}(\mathbf{q}_2)}{\Omega_{s_1}(\mathbf{q}_1)}\Big[\sum_{\mathbf{q}_3s_3}[\Gamma^{(3)}_{s_1s_2s_3}(\mathbf{q}_1,\mathbf{q}_2,\mathbf{q}_3,\Omega_{s_1}(\mathbf{q}_1)) + \Gamma^{(3)}_{s_1s_2s_3}(\mathbf{q}_1,\mathbf{q}_3,\mathbf{q}_2,\Omega_{s_1}(\mathbf{q}_1))] \\ & + \Gamma^{iso}_{s_1s_2}(\mathbf{q}_1,\mathbf{q}_2,\Omega_{s_1}(\mathbf{q}_1))\Big],\end{aligned} \tag{S34}$$

where $\Gamma^M_s(\mathbf{q}) = \Gamma_s(\mathbf{q},\Omega_s(\mathbf{q}))$.

In the limit where the quasi-particle concept is still valid, the off-diagonal component of the thermal conductivity tensor κ^{od} can be expressed as [32]

$$\kappa^{od} = \frac{1}{V}\sum_{\mathbf{q}}{\sum_{s_1s_2}}' \mathbf{v}^{s_1s_2}_{\mathbf{q}}\otimes\mathbf{v}^{s_1s_2}_{\mathbf{q}}\frac{c^{s_1}_{\mathbf{q}}+c^{s_2}_{\mathbf{q}}}{2}\Gamma^M_{s_1s_2}(\mathbf{q}), \tag{S35}$$

where the off-diagonal scattering $\Gamma^M_{s_1s_2}$ is given by

$$\Gamma^M_{s_1s_2}(\mathbf{q}) = \frac{\Gamma^M_{s_1}(\mathbf{q})+\Gamma^M_{s_2}(\mathbf{q})}{[\Omega_{s_1}(\mathbf{q})-\Omega_{s_2}(\mathbf{q})]^2+[\Gamma^M_{s_1}(\mathbf{q})+\Gamma^M_{s_2}(\mathbf{q})]^2}. \tag{S36}$$

In this formalism, ${\sum}'_{s_1s_2}$ indicates that $s_1 = s_2$ is not taken into account.

We emphasize that this off-diagonal term captures wave-like interference between phonons of similar frequencies and can significantly contribute to the thermal conductivity tensor in materials with complex structures [36]. We mention that in our thermal conductivity calculations, a $\mathbf{q}$-mesh of $9\times9\times9$ points was employed. Finer $\mathbf{q}$-point meshes were not employed due to their prohibitive computational cost. The calculated values of κ were obtained iteratively.

In summary, the strength of phonon scattering in our theoretical model is governed by the third-order IFCs and the isotopic distribution in BBO. The former is expected to provide a substantial contribution due to the material's anharmonicity and the large number of allowed three-phonon processes.

---

[1] D. Cahill, Thermal conductivity measurement from 30 to 750 K: the 3ω method, Review of scientific instruments **61**, 802 (1990).

[2] A. Henriques, M. Santoma, S. Wirth, J. Larrea Jiménez, and V. Martelli, Two lock-in amplifiers based 3ω technique: a practical guide for thermal conductivity experiments in bulk samples (2025), arxiv.org/abs/2504.21820.
[3] D. A. Keen, M. J. Gutmann, and C. C. Wilson, SXD - the single-crystal diffractometer at the ISIS spallation neutron source, Journal of Applied Crystallography **39**, 714 (2006).
[4] V. Petříček, M. Dušek, and L. Palatinus, Crystallographic computing system jana2006: General features, Zeitschrift für Kristallographie - Crystalline Materials **229**, 345 (2014).
[5] S. Parsons, Introduction to twinning, Acta Crystallographica Section D **59**, 1995 (2003).
[6] O. Foyevtsov, S. Balandeh, S. Chi, and G. Sawatzky, Structural electronic and magnetic properties of $BaBiO_3$ single crystals, Physica B: Condensed Matter **570**, 328 (2019).
[7] R. Kuentzler, C. Hornick, Y. Dossman, S. Wegner, R. El Farsi, and M. Drillon, Superconductivity of Pb, K and Rb-doped $BaBiO_3$, Physica C: Superconductivity **184**, 316 (1991).
[8] O. L. Anderson, A simplified method for calculating the debye temperature from elastic constants, Journal of Physics and Chemistry of Solids **24**, 909 (1963).
[9] J. P. Poirier, Lindemann law and the melting temperature of perovskites, Physics of the earth and planetary interiors **54**, 364 (1989).
[10] T. Shang, A. Amon, D. Kasinathan, W. Xie, M. Bobnar, Y. Chen, A. Wang, M. Shi, M. Medarde, H. Q. Yuan, and T. Shiroka, Enhanced tc and multiband superconductivity in the fully-gapped $ReBe_22$ superconductor, New Journal of Physics **21**, 073034 (2019).
[11] J. P. Perdew, K. Burke, and M. Ernzerhof, Generalized gradient approximation made simple, Phys. Rev. Lett. **77**, 3865 (1996).
[12] P. E. Blüchl, Projector augmented-wave method, Phys. Rev. B **50**, 17953 (1994).
[13] G. Kresse and J. Furthmüller, Efficiency of ab-initio total energy calculations for metals and semiconductors using a plane-wave basis set, Comput. Mater. Sci. **6**, 15 (1996).
[14] G. Kresse and J. Furthmüller, Efficient iterative schemes for ab initio total-energy calculations using a plane-wave basis set, Phys. Rev. B **54**, 11169 (1996).
[15] A. Togo, L. Chaput, T. Tadano, and I. Tanaka, Implementation strategies in phonopy and phono3py, J. Phys. Condens. Matter **35**, 353001 (2023).
[16] A. Togo, First-principles phonon calculations with phonopy and phono3py, J. Phys. Soc. Jpn. **92**, 012001 (2023).
[17] R. Jinnouchi, J. Lahnsteiner, F. Karsai, G. Kresse, and M. Bokdam, Phase transitions of hybrid perovskites simulated by machine-learning force fields trained on the fly with bayesian inference, Phys. Rev. Lett. **122**, 225701 (2019).
[18] R. Jinnouchi, F. Karsai, and G. Kresse, On-the-fly machine learning force field generation: Application to melting points, Phys. Rev. B **100**, 014105 (2019).
[19] R. Jinnouchi, F. Karsai, C. Verdi, R. Asahi, and G. Kresse, Descriptors representing two- and three-body atomic distributions and their effects on the accuracy of machine-learned inter-atomic potentials, The Journal of Chemical Physics **152**, 234102 (2020), https://pubs.aip.org/aip/jcp/article-pdf/doi/10.1063/5.0009491/15575269/234102_1_online.pdf.
[20] O. Hellman, P. Steneteg, I. A. Abrikosov, and S. I. Simak, Temperature dependent effective potential method for accurate free energy calculations of solids, Phys. Rev. B **87**, 104111 (2013).
[21] O. Hellman and I. A. Abrikosov, Temperature-dependent effective third-order interatomic force constants from first principles, Phys. Rev. B **88**, 144301 (2013).
[22] O. Hellman and D. A. Broido, Phonon thermal transport in Bi_2Te_3 from first principles, Phys. Rev. B **90**, 134309 (2014).
[23] A. H. Romero, E. K. U. Gross, M. J. Verstraete, and O. Hellman, Thermal conductivity in PbTe from first principles, Phys. Rev. B **91**, 214310 (2015).
[24] J. P. Watt, Hashin-Shtrikman bounds on the effective elastic moduli of polycrystals with monoclinic symmetry, Journal of Applied Physics **51**, 1520 (1980).
[25] Z.-j. Wu, E.-j. Zhao, H.-p. Xiang, X.-f. Hao, X.-j. Liu, and J. Meng, Crystal structures and elastic properties of superhard IrN_2 and IrN_3 from first principles, Physical Review B **76**, 054115 (2007).
[26] J. Fu, Elastic constants and homogenized moduli of monoclinic structures based on density functional theory, Density Functional Calculations: Recent Progresses of Theory and Application **219** (2018).
[27] V. Wang, N. Xu, J.-C. Liu, G. Tang, and W.-T. Geng, Vaspkit: A user-friendly interface facilitating high-throughput computing and analysis using vasp code, Computer Physics Communications **267**, 108033 (2021).
[28] J. Gao, W. Zeng, B. Tang, M. Zhong, and Q.-J. Liu, Optical, electronic, and mechanical properties of p-type conductive oxide $BaBiO_3$: A density functional theory study, Chemical Physics Letters **761**, 138054 (2020).
[29] Z. Feng, Y. Fu, Y. Zhang, and D. J. Singh, Characterization of rattling in relation to thermal conductivity: Ordered half-Heusler semiconductors, Phys. Rev. B **101**, 064301 (2020).
[30] A. Togo, L. Chaput, I. Tanaka, and G. Hug, First-principles phonon calculations of thermal expansion in Ti_3SiC_2, Ti_3AlC_2, and Ti_3GeC_2, Phys. Rev. B **81**, 174301 (2010).
[31] A. Castellano, J. P. A. Batista, and M. J. Verstraete, Mode-coupling theory of lattice dynamics for classical and quantum crystals, The Journal of Chemical Physics **159**, 234501 (2023).
[32] A. Castellano, J. P. A. Batista, O. Hellman, and M. J. Verstraete, Mode-coupling formulation of heat transport in anharmonic materials, Phys. Rev. B **111**, 094306 (2025).
[33] A. A. Maradudin, A. E. Fein, and G. H. Vineyard, On the evaluation of phonon widths and shifts, physica status solidi (b) **2**, 1479 (1962), https://onlinelibrary.wiley.com/doi/pdf/10.1002/pssb.19620021106.
[34] S.-i. Tamura, Isotope scattering of dispersive phonons in Ge, Phys. Rev. B **27**, 858 (1983).
[35] G. Fugallo, M. Lazzeri, L. Paulatto, and F. Mauri, Ab initio variational approach for evaluating lattice thermal conductivity,

Phys. Rev. B **88**, 045430 (2013).
[36] G. Caldarelli, M. Simoncelli, N. Marzari, F. Mauri, and L. Benfatto, Many-body green's function approach to lattice thermal transport, Phys. Rev. B **106**, 024312 (2022).
